# Lipoprotein(a) beyond the kringle IV repeat polymorphism: The complexity of genetic variation in the *LPA* gene

**DOI:** 10.1016/j.atherosclerosis.2022.04.003

**Published:** 2022-05-01

**Authors:** Stefan Coassin, Florian Kronenberg

**Affiliations:** Institute of Genetic Epidemiology, Medical University of Innsbruck, Schoepfstrasse 41, 6020, Innsbruck, Austria

**Keywords:** Lipoprotein(a), Genetics, Kringle IV polymorphism, LPA, Ancestry, Ethnicity, Kringle IV-2

## Abstract

High lipoprotein(a) [Lp(a)] concentrations are one of the most important genetically determined risk factors for cardiovascular disease. Lp(a) concentrations are an enigmatic trait largely controlled by one single gene (LPA) that contains a complex interplay of several genetic elements with many surprising effects discussed in this review. A hypervariable coding copy number variation (the kringle IV type-2 repeat, KIV-2) generates >40 apolipoprotein(a) protein isoforms and determines the median Lp(a) concentrations. Carriers of small isoforms with up to 22 kringle IV domains have median Lp(a) concentrations up to 5 times higher than those with large isoforms (>22 kringle IV domains). The effect of the apo(a) isoforms are, however, modified by many functional single nucleotide polymorphisms (SNPs) distributed over the complete range of allele frequencies (<0.1% to >20%) with very pronounced effects on Lp(a) concentrations. A complex interaction is present between the apo (a) isoforms and *LPA* SNPs, with isoforms partially masking the effect of functional SNPs and, vice versa, SNPs lowering the Lp(a) concentrations of affected isoforms. This picture is further complicated by SNP-SNP interactions, a poorly understood role of other polymorphisms such as short tandem repeats and linkage structures that are poorly captured by common R^2^ values. A further layer of complexity derives from recent findings that several functional SNPs are located in the KIV-2 repeat and are thus not accessible to conventional sequencing and genotyping technologies. A critical impact of the ancestry on correlation structures and baseline Lp(a) values becomes increasingly evident.

This review provides a comprehensive overview on the complex genetic architecture of the Lp(a) concentrations in plasma, a field that has made tremendous progress with the introduction of new technologies. Understanding the genetics of Lp(a) might be a key to many mysteries of Lp(a) and booster new ideas on the metabolism of Lp(a) and possible interventional targets.

## 1 Lipoprotein(a) plasma concentrations – an enigmatic trait

Lipoprotein(a) [Lp(a)] has proatherogenic, proinflammatory and possibly prothrombotic properties and represents a major cardiovascular risk factor in the general population [1,2]. Atherogenic Lp(a) concentrations affect up to 2 billion people worldwide [3] (>30 [1] or >50 [4] mg/dL). Several details of the pathophysiology of Lp(a) are not fully clarified but converging data proposes that the proinflammatory oxidized phospholipids (OxPLs) play a key role in mediating several detrimental effects of Lp(a) [5] as discussed in another review of this series [6].

**Fig. 1:**
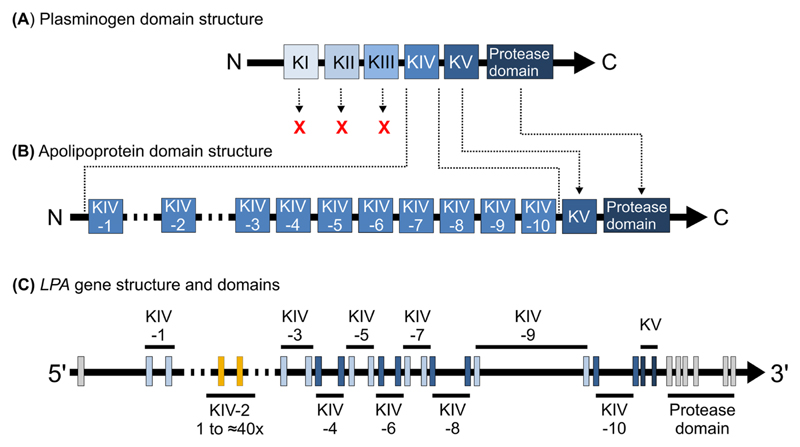
*LPA* evolution from plasminogen and the respective domain and gene structures. (A) Plasminogen domain structure consisting of five kringle domains (I to V) and a C-terminal protease domain. (B) Apolipoprotein(a) domain structure. The origin of the domains from their precursors in plasminogen (A) is shown by arrows. *LPA* originated from plasminogen by gene duplication, loss of KI to KIII, expansion of KIV, introduction of a CNV structure for the KIV-2, and retaining of KV and the protease domain (which was inactivated by mutations). (C) Gene structure of *LPA,* with every kringle consisting of two short exons, spaced by a mostly ≈4 kb large intron (except KIV-9, 19 kb). A ≈1.2 kb intron separates the KIV units. The start of exon 1 has changed over time, with some early studies using an annotation with 90 additional bases on the 5’ side [66,88,101]. Ensembl annotations using the human genome reference GRCh37/hg19 and NCBI36/hg18 (before release 76; ENST00000447678.1) contained an additional non-coding exon ≈4 kb upstream of the current exon 1. This was not present in the very first genetic studies and has been removed again in the current annotations.

The Lp(a) particle originates from the liver, is found only in old world monkeys and apes [7] and consists of an apolipoprotein(a) [apo (a)] molecule that is bound to the apoB100 moiety of an LDL-like lipoprotein [7]. *LPA,* the gene encoding apo(a), evolved from a duplication of the plasminogen (*PLG*) gene about 33–40 million years ago [8,9]. While plasminogen kringle domains I, II and III (KI, KII, KIII) were lost, the KIV domain expanded and diverged to 10 subtypes (KIV-1 to KIV-10) [7]. Kringle V and the protease domain were retained but the protease domain was inactivated by mutations [8] (Fig. 1). The KIV-2 domain is encoded by a copy number variation (CNV) that creates >30 gene alleles, respectively protein isoforms (≈200–800 kDa) in the population [10–15]. By convention, apo(a) isoform designations report the total number of KIV domains [16]. The KIV-2 number can be deduced by subtracting nine kringles.

**Fig. 2:**
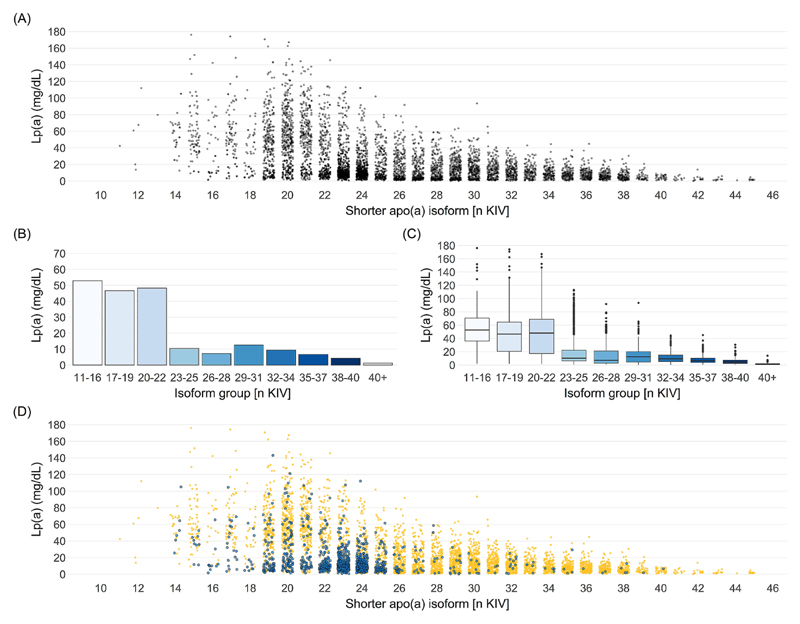
Lp(a) variance in a general European population. (A) Lp(a) concentrations in each isoform group (defined in heterozygotes by the smaller isoform present). This shows the large variance of Lp(a) within each isoform group. Many samples with very low Lp(a) can be observed in each apo(a) isoform group, being most pronounced in isoforms 23 and 24. This is caused largely by the variant KIV-2 4925G>A (discussed in the section about KIV-2 variants), as well as partially by KIV-2 4733G>A [30] and other variants. (B) Median Lp(a) in isoforms groups (groups according to Ref. [1]). The concentrations decrease sharply between 22 and 23 KIV. (C) Box plots of the same data as in panel B shows a considerable variance in each group. Data are often shown in the literature as in Panel B which ignores the enormous variability in each apo(a) isoform group. (D) Same figure as panel A, but with the carriers of KIV-2 4925G>A shown in blue (yellow: non-carriers). This shows well how a strongly Lp(a)-modifying SNP may cluster with a defined isoform range. Several similar examples are described in Refs. [24,38]. Data is from the general population studies KORA [146] F3 and F4 (n = 5807 in panel A and D n = 6005 in panels B and C; updated from Ref. [29]). Study design and Lp(a) phenotyping have been described in Refs. [29,77,85].

Lp(a) is one of the most heritable human quantitative traits with up to ≈90% heritability [10,17]. Individual Lp(a) concentrations are relatively stable throughout life-time (albeit some currently underappreciated temporal variability exits [18]). Lp(a) distribution is highly right-skewed in Whites with medians of ≈10–12 mg/dL [1]. The inter-individual Lp(a) concentrations extend for about three orders of magnitude (<0.1 mg/dL to >300 mg/dL) [1] (Fig. 2A) and show considerable cross-ancestry variance [19] (reviewed by Virani and colleagues in this review series [20]). In Black populations, the distribution is more Gaussian and median concentrations are markedly higher [19,21,22]. It has been suggested that genetic variability might be responsible for some of the cross-ancestry differences in Lp(a) concentrations [23,24].

While >160 genes are necessary to explain 50–70% of the heritability of other lipoproteins [25,26], the *LPA* gene locus alone explains up to 90% of Lp(a) variance [10,17]. About 40–70% of Lp(a) variance is explained by the apo(a) isoform size, which shows an inverse relationship with the Lp(a) concentration [1,14] (Fig. 2A). This is most probably due to a more efficient maturation of smaller apo(a) proteins in the endoplasmatic reticulum [27,28]. Low molecular weight (LMW) isoforms (10–22 KIV) are associated with ≈4–5 times higher median Lp(a) concentrations (≈40–50 mg/dL) than high molecular weight (HMW; >22 KIV) isoforms (<10 mg/dL) [1] and the concentrations decrease rather suddenly at 23 KIV [1] (Fig. 2B). These considerably higher median Lp(a) concentrations in LMW individuals have been well established since a long time. However, the exact relationship between apo(a) isoform and Lp(a) concentrations is complex, not linear and modified also by several functional single nucleotide polymorphisms (SNPs) (discussed in depth in the following sections). These lead to a large (yet in the field still underappreciated) variance in the individual Lp(a) concentrations within each isoform group (Fig. 2C). Fig. 2D provides an example of such a strongly Lp(a)-modifying SNP (KIV-2 4925G>A) [24,29,30]. Because of the differing maturation efficiencies and the modifying SNPs, the two *LPA* alleles of heterozygous individuals are not necessarily equally secreted to plasma. The relative contribution can be visualized by Western blotting and used to apportion the plasma Lp(a) concentration to isoform-specific Lp(a) concentrations [31,32], nuancing the Lp(a) trait further.

## 2 Why are we keen to understand the genetic regulation of Lp(a)?

Understanding the genetics of Lp(a) might be a key to many mysteries of Lp(a) (Key point box 2).

1. Genetic variants associated with certain Lp(a) concentration ranges were already in the past very helpful to support causality between Lp(a) and outcomes. Lp(a) was the first use case for Mendelian randomization studies in the 1990s [33,34], long before this term was coined (see review [35] in this series). Nevertheless, the causality of Lp(a) has been debated for a long time [36] until numerous genetic studies underscored the causality of Lp(a) concentrations using genetic variants strongly associated with high Lp(a) concentrations and subsequent cardiovascular disease [37–44]. Several studies showed also that variants associated with low Lp(a) exert a protective effect on cardiovascular disease [29,30,38,45,46]. On the other hand, it still takes some efforts to find the right genetic instrument to investigate a causal association between extremely low Lp(a) concentrations and diabetes mellitus [38,47–49]. In the latter case, the use of the non-carrier status of rs10455872 is an insufficient genetic instrument, as discussed earlier [49] and in this review series by Lamina and Ward [50]: it comprises around 85% of the population and therefore includes the majority of the Lp(a) concentration distribution, including a substantial fraction of the population with high Lp(a) concentrations as well as large and small apo(a) isoforms.
2. Lp(a) concentrations show pronounced differences across ancestries, which we do not completely understand yet. There is evidence that SNPs with a strong influence on Lp(a) concentrations show a wide frequency variability across ancestries [24,29,30,51]. Others also suggested a role of environmental exposures, respectively differing inflammatory burden [52,53]. Identifying the unknowns might bring us closer to the full picture of cross-ancestry genetic regulation of the Lp(a) concentration differences, helping to dissect the relative contribution of genetics and environment in determining the Lp(a) trait across ancestries.
3. We have currently a limited understanding of the metabolism and especially the catabolism of Lp(a). Searching for Lp(a)-regulating SNPs outside the *LPA* gene region might provide new evidence on genes involved in the machinery of Lp(a) catabolism. On the other hand, such studies are complicated by the considerable variance encoded by *LPA.* A successful search for modulators of Lp(a) metabolism outside of the *LPA* locus may require identifying individuals with peculiar Lp(a) concentrations that are not caused merely by different *LPA* SNPs. However, this requires a comprehensive knowledge about the functional *LPA* SNPs. Interestingly, such focused in-depth genetic studies on peculiar Lp(a) phenotypes are still rare [54].
4. Genetic variants and how they regulate Lp(a) concentrations could identify targets for future drug interventions. As individuals with low Lp(a) do not present obvious health impairments [46,55], *LPA* is a clear early candidate for the therapeutic intervention using somatic gene editing [56]. On the dawn of these therapies, frequent Lp(a)-lowering variants may indicate possible genome locations for safe gene editing. Conversely, the identification of regulatory polymorphisms that do increase Lp(a) rather than lower it can be highly informative as well, indicating targets that could be addressed by e.g. inhibitors.

## 3 Structure of the *LPA* gene

*LPA* spans a >130 kb region on chromosome 6 (160,531,483–160,666,375 in the current human genome reference sequence hg38). Its evolutionary history produced a fairly regular and repetitive gene structure (Fig. 1C) with extensive intragenic and intergenic homologies. Each kringle consist of two short exons (160 and 182 bp long, except for KIV-6) highly homologous to the respective exons of the other kringles [7] (>70% base identity between the different KIV; 98–100% between the KIV-2 exons [57]). This homology extends into the intronic sequences with often >60% base identity in the first ±200 intronic bases (Supplemental Fig. 1). Furthermore, *LPA* is highly homologous to the (i) often neglected liver-expressed pseudogene LPA-like 2 (*LPAL2*) [58], which flanks *LPA* upstream and contains sequences homologous to KIV-2, KIV-9, KIV-10 and the protease domain, (ii) plasminogen, which flanks *LPA* downstream and (iii) the plasminogen pseudogenes *PLGLA, PLGLB1, PLGLB2* on chromosome 2. The *LPA* KIV and KV introns contain large, mostly quite diverged insertions of long interspersed nuclear element-1 retrotransposons (L1 LINEs). L1 LINEs are frequent mobile genetic elements that make up ≈ 20% of the human genome. They may induce structural variation by providing hotspots for recombination events and/or affect gene expression by containing regulatory elements [59,60]. However, the specific significance of these intronic L1 elements for Lp(a) concentrations (if any) has not been explored yet.

The KIV-2 CNV presents ≈40 different alleles, resulting in ≈1600 possible genotypes [10–12,61]. This makes up to 70% of the gene hardly accessible for mutation detection and to an uncharted territory on the human genetic map. Each KIV-2 repeat is ≈ 5550 bp long, with some minor variability created by small indels [57] and an intronic short tandem repeat (STR) [62]. The haplotype of three synonymous SNPs in KIV-2 exon 1 defines at least three types of KIV-2 units (KIV-2A, KIV-2B and KIV-2C; Supplemental Fig. 2) [8,63,64], which differ also by >100 intronic differences [57]. One such difference splits KIV-2A in two subtypes [24]. No effect of these subtypes on Lp(a) concentrations was observed [57], but they have practical implications for research studies using next generation sequencing (NGS) (explained below). The current human genome reference sequence hg38 contains only six KIV-2 repeats (the third being KIV-2B). This creates considerable alignment difficulties for NGS data, which may contain up to 40 KIV-2 repeats.

Different ancestries differ in the minor allele frequency (MAF) of these subtypes and in the percentage of KIV-2 repeats being type B or C. The resulting mutation level [57] in the NGS data (also called intra-allelic frequency [64]) ranges from <5% in Africans to >30% in East Asians [57,64]. Within Europe, the low KIV-2B intra-allelic frequencies in Southern Europe and higher frequencies in Finns correlate well with the known intra-European Lp(a) gradient [65], likely reflecting differences in the genetic ancestry at the *LPA* locus.

## 4 Regulation of *LPA*

The transcriptional regulation of *LPA* is not fully understood, but involves proximal and distal elements. A 200 bp core promoter region is sufficient to drive *LPA* expression [66], but the overall activity of promoter fragments encompassing up to 1.5 kb 5’ region is low [67,68]. Many transcription factors have been described to regulate *LPA* transcription [69] but few have been validated in independent studies and/or by identification of the response elements. Additionally, multiple transcription factor modules with opposing effects have been reported in the promoter [70] and functional promoter elements have been identified as far as ≈3.5 kb from the currently annotated transcription start site [71]. *In vitro* activities may thus strongly depend on the assayed region [70].

*LPA* is regulated also by two enhancer elements [72,73] located ≈20 kb (region DH-III; chr6:160683862-160685654 [73], hg38) and ≈30 kb (region DH-II; chr6: 160692643–160694671 [73], hg38) upstream. They contain multiple response elements (Sp1 [73], PPAR factors [73] and estrogen [74]) and induce the *LPA* promoter by 4–8 and 10–15 fold, respectively [73]. Despite DH-II has a smaller effect on *LPA* transcription than DH-III, early population studies reported that it is more conserved than DH-III [75,76], but no systematic studies in large, more recent genetic resources have been reported yet. Intriguingly, the SNP rs186696265, which has the largest independent beta estimate (i.e. the effect on Lp(a) concentrations in mg/dL or nmol/L) identified so far but a low MAF (1.5%), is located between DH-II and DH-III. It increases Lp(a) by 49 mg/dL in an isoform-adjusted model and the odds for coronary artery disease (CAD) by 1.73-fold [77]. This SNP has been identified by multiple GWAS on Lp(a) concentrations, plasma cholesterol phenotypes, triglycerides, cardiovascular phenotypes and even longevity [78] but no function has been assigned yet.

## 5 Dissociation of apo(a) isoform size and Lp(a) concentrations

The isoform alone is not predictive of the Lp(a) concentration in a given individual [14]. At individual level, same-sized isoforms may be associated with 200-fold different Lp(a) values [17,79]. This can be seen in Fig. 2A, demonstrating that the range in Lp(a) concentrations, e.g. in carriers of small isoforms with 20 KIV repeats, is ranging from below 1 mg/dL to almost 200 mg/dL. This is observed also in individuals expressing only one isoform, indicating that this variance is not caused only by the often neglected contribution of the second isoform [80,81]. Conversely, the inter-individual variation of alleles that are identical-by-descent within families is markedly smaller (only up to 3-fold) [79]. This implies that other genetic variants exist, which dissociate the commonly assumed inverse relationship between apo(a) isoform size and Lp(a) concentrations in a substantial manner. Especially early studies reported and characterized many examples of such “discordant phenotypes” [11,17,79–83]. For example, Cohen et al. [82] described a family where two same-sized alleles (23 KIV) that were distinguishable by an intronic SNP segregated with strikingly different concentrations (1–3 mg/dL vs. ≈22 mg/dL). Such discordant phenotypes can be seen quite often in small apo(a) isoform carriers who have low Lp(a) concentrations despite their small apo(a) isoform [29,30,83]. This additional modification of the isoform effect is mirrored across populations and ancestries, with Africans showing much higher Lp(a) concentrations in every isoform group [21,84] and Finns showing 50% lower Lp(a) in every isoform group than Central Europeans [85].

Causal variants have been elusive for a long time. However, studies on an STR in the promoter (known as the **“pentanucleotide repeat (PNR)”**; hg38 chr6:160,665,587–160,665,631) provided interesting insights into the diversity of these phenotypes. The different PNR alleles with ≈6–12 repeat units (PNR6 to PNR12) [83] are associated with very diverse Lp(a) phenotypes. PNR8 alleles are the most frequent ones and recapitulate the full range of Lp(a) and isoform correlations [83]. PNR9 alleles are the human genome reference allele and occur mostly in the HMW isoform range [83]. PNR10 alleles show two different subgroups: one subgroup follows the expected correlation of isoform and Lp(a) across the whole isoform range, while the second subgroup tags LMW isoforms with low Lp(a) (<≈15 mg/dL) [83]. Finally, PNR11 alleles tag isoforms with <24 KIV but unexpected low Lp(a) <5 mg/dL [83]. The causal factors for the discordant phenotypes tagged by PNR10 and PNR11 have not been identified yet.

Similar observations were made also with a DraIII restriction polymorphism found in only some KIV-2 units (KIV-D) [86]. The order of KIV-2 and KIV-D units creates nearly 30 individual restriction patterns, which likely mark different background haplotypes [86]. Patterns 3 and 4 were linked to very defined isoform ranges (pattern 3: 27–32 KIV, pattern 4: 27–29 KIV), were associated with very low Lp(a) concentrations, and encompassed 24% and 6% of all null alleles in the study population [86]. Our group recently identified the base change underlying the DraIII restriction polymorphism, but the SNP alone was not able to act as a proxy for these complex restriction patterns [57].

Overall, these and other studies indicate the existence of a large diversity of haplotypes that are restricted to certain isoform ranges and are associated with very distinct Lp(a) concentrations [82,86–89]. Some causal variants are discussed below.

## 6 Shaping of the Lp(a) trait by genetic variants

**Fig. 3:**
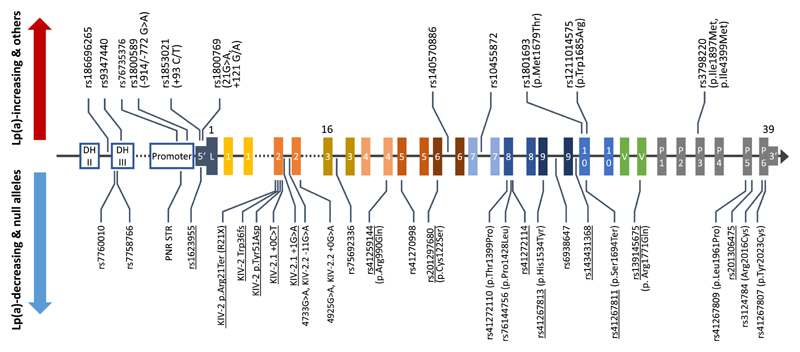
Location of relevant *LPA* SNPs. Location of multiple *LPA* SNPs with remarkable effects that have been discussed in the literature. Table 1 provides background information. The exons are numbered according to the domain that they encode (1-10: KIV-1 to KIV-10, L. leader sequence, P. protease domain, 5’: 5’UTR, 3’: 3’ UTR). For orientation, some exons carry a superscript reporting the exon number in the genome sequence hg38. SNPs that have been associated with increased Lp(a) concentrations or that act through other mechanisms (rs1211014575, which prevents OxPL binding) are shown above the gene structure; SNPs that have been associated with decreased Lp(a) (both causally or by association only) are shown below. SNPs that cause null alleles are underlined, albeit many more Lp(a)-lowering SNPs may cause null alleles if occurring on an allele with already low Lp(a) production. SNPs in the KIV-2 are named according to their publication, as they cannot be assigned a single rs-identifier because their location is not unique. Gene structure is not in scale.

**Fig. 4:**
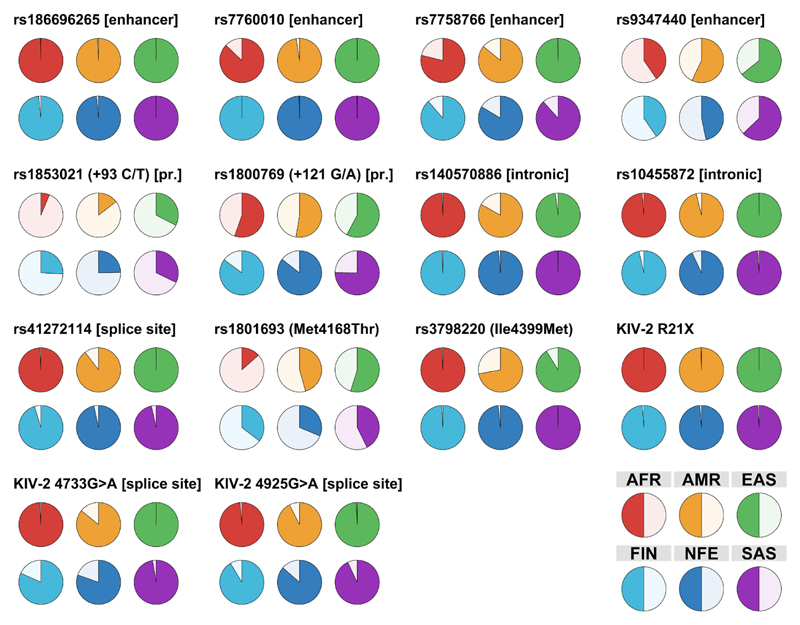
Minor allele frequencies of selected *LPA* SNPs that are assumed or confirmed to be functional. Several assumed or confirmed functional LPA SNPs show considerable MAF differences between population and ancestries. Selected SNPs are shown in this figure. Frequencies are from gnomAD [116] exome data v 2.1.1 for coding SNPs (125,748 exomes, 15,708 genomes) and from gnomAD 3.1.2 (76,156 genomes) for non-coding SNPs. For the KIV-2 SNPs 4733G>A [30], 4925G>A [29] and R21X [96], the MAF was estimated from the carrier frequency reported in the respective publications (which were based on the 1000 Genomes phase 3v5 [147] sequencing data, n = 2504 genomes) assuming Hardy-Weinberg-equilibrium. Light color indicates the minor allele according to the human genome hg38. Note that this is not necessarily the effect allele of the single SNPs (for example for rs1853021). The population color code is given bottom-right. Population codes are from GnomAD: AFR: African/African American, AMR: Latino/Admixed American, EAS: East Asian, FIN: European (Finnish), NFE: European (non-Finnish), SAS: South Asian. For non-missense SNPs, a description is given in square bracket for better classification (pr.: promoter).

Many studies have aimed at identifying SNPs that causally affect Lp(a) concentrations. Since an individual discussion of all interesting *LPA* SNPs that have been described would go far beyond the focus of this review, Table 1 provides a comprehensive summary for those of particular interest and characterization. Fig. 3 and Fig. 4 report location and MAFs of selected SNPs.

**Fig. 5:**
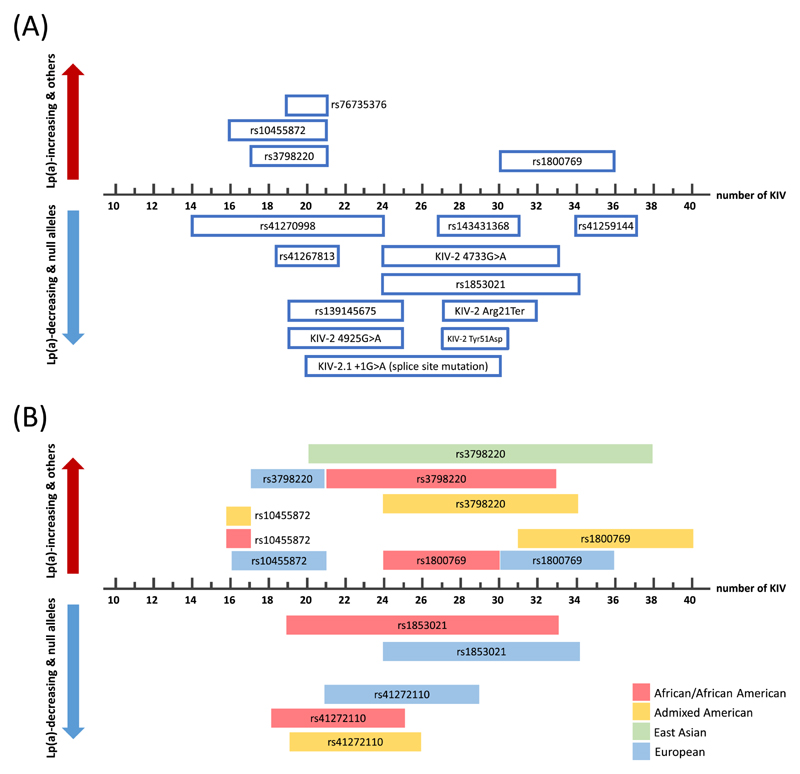
Association of SNPs with apolipoprotein(a) isoforms. (A) Association of selected SNPs with given apo(a) isoform ranges in Europeans, stratified by Lp(a)-increasing or Lp(a)-decreasing variants, as in Fig. 3. This shows considerable differences across SNPs. (B) Association of selected SNPs with different isoform ranges across ancestries (ancestry color code given bottom-right). Unfortunately, this data is available for only very few SNPs, but notable differences can be appreciated, which can bias cross-ancestry studies. Note that no truly structured and standardized data is available. For most SNPs isoform-association has been assessed only by one or maximum a few studies. Therefore, this figure has been assembled from multiple technologies such as LPA genotyping by pulsed-field gel electrophoresis [10,11], Western blotting and imputed KIV-2 content [24]. The ranges given here are thus purely indicative and, especially at single individual level, association with other isoforms may be possible as well. When various overlapping ranges were reported by different authors, the widest range is shown. Additional information and references are given in Table 1. For simplicity, boxes with defined boundaries have been used for representation (the limits are based on literature reports), but for many SNPs the isoform-association is not that well confined and extends also beyond the limits given here. For example, KIV-2 4733G>A is seen predominantly in 24–33 KIV but found across the whole isoform range.

**Fig. 6:**
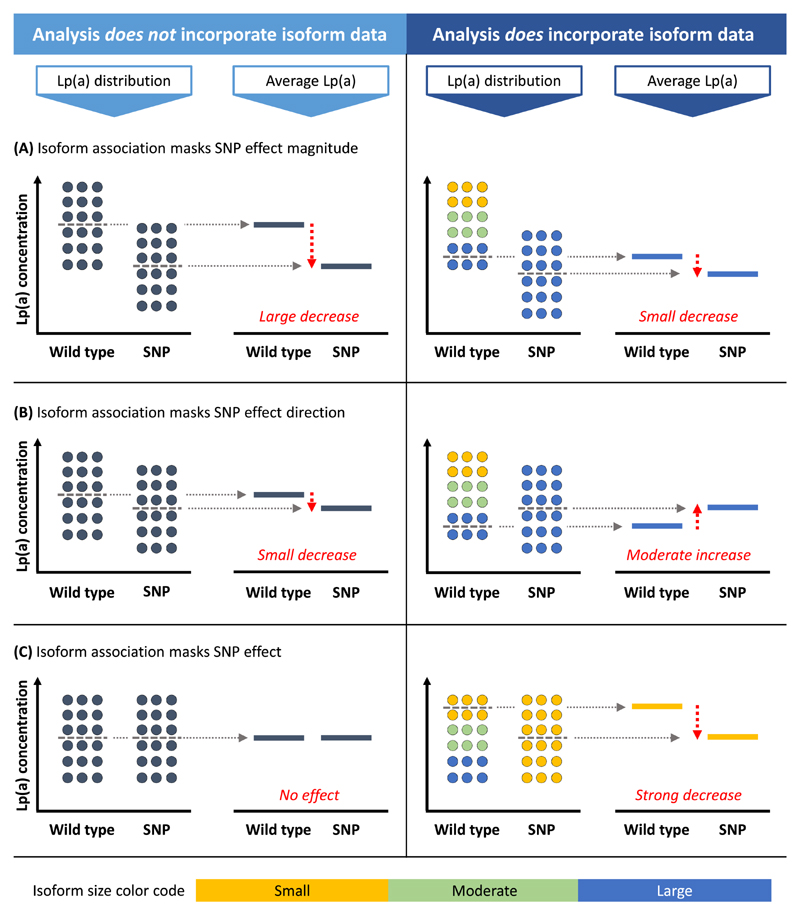
The background isoform affects the interpretation of *LPA* SNP (selected examples). The association of *LPA* SNPs with defined isoform ranges can mask their true effect. This figures describes three basic principles but several other combinations are possible, and each example could also be conceived into the opposite direction. For better representation, we assume a simplified trait with three well-defined isoform ranges clearly associated with high, moderate and low Lp(a) concentrations, respectively). Each SNP is associated only with one range. The exemplary SNPs affect the average Lp(a) concentrations in the groups but not the Lp(a) variance. The second isoform is omitted for simplicity. The **left side of the** figure describes the effect observed when just comparing wild type and SNP carriers (i.e. carriers of the variant base). This analysis reflects the analyses that are performed in common SNP association studies. The left panel shows the distribution of 18 exemplary individuals per group, with the y-axis representing the Lp(a) concentrations. Every dot represents an individual. The right panel shows the location of the respective average Lp(a) values. The red arrow indicates the resulting SNP effect. The **right side of the figure** shows the same data, but color-coded for the background isoform. The incorporation of the isoforms into the analysis changes the reference average. This can mitigate (example A), reverse (example B) or unmask (example C) the real effect of a SNP. It is important to note that, depending on the aim of the study, both types of analyses may actually be “correct”. Unadjusted analyses capture indirectly also the effect of the isoforms and may be appropriate for general association studies or construction of genetic risk scores. Isoform-adjusted studies can identify SNPs that govern Lp(a) variance in subgroups, improving the overall variance explained, and help to develop hypotheses for functional studies. See the main text for discussion of the SNP mentioned as examples. (**A, left side (SNP only)**) SNP variant is associated with low Lp(a). (**A, right side (background isoform considered)**) this SNP is located on large apo(a) alleles with a low expression level. This limits the total SNP effect. Examples: rs1853021, rs41272114. (**B, left**) An SNP is associated with low Lp(a). (**B, right**) This SNP is actually associated with increased Lp(a) but it is located on large isoforms. The overall Lp(a)-lowering effect of the large isoforms masks the Lp(a)-increasing effect of the SNP. Example: rs1800769. (**C, left**) The SNP has no effect on Lp(a). (**C, right**) When considering that this SNP is located on short isoforms, the SNP becomes strongly Lp(a)-decreasing. Example: KIV-2 4925G>A.

The relationship between SNPs and Lp(a) concentrations is not always straight-forward. Multiple, partially very recent, studies have provided fascinating insights into the complex genetic entanglements that govern the Lp(a) concentrations. These involve allelic association between SNPs and isoform ranges [23,39,51,88,90,91] (Fig. 5), between different SNPs [88,92–94] and between SNPs and STRs [87,88] and span the complete gene body [88,93,95]. They can confound associations [29,51,96] and may even reverse the direction of an SNP effect (Fig. 6). The following sections will discuss some SNPs that exemplify these entanglements particularly well. Importantly, nearly all these SNPs affect directly Lp(a) concentrations. A noteworthy exception is **rs1211014575** (KIV-10 Trp72Arg [97], p.Trp1685Arg), which abolishes the lysine binding capacity of KIV-10 [97,98] without affecting Lp(a) concentrations [99], preventing OxPL accumulation on KIV-10 [5] (Table 1).

## 7 Association of SNPs and apo(a) isoforms

Many *LPA* SNPs are restricted to specific isoform ranges (Table 1 and Fig. 5A). This can enhance, limit or even mask the effects of functional SNPs. A clear-cut loss-of-function (LOF) mutation on an HMW allele may contribute little to the total plasma Lp(a) concentration since the concentration connected with an HMW allele is already low [96]. Otherwise, a moderate LOF mutation on an LMW allele can have considerable effects [29]. Furthermore, the overall expression level of the isoform may mask an opposite effect of an SNP [81,100] (Fig. 6). The two regulatory SNPs rs1853021 and rs1800769 exemplify this particularly well. Further prime examples are described in the subsequent sections about the KIV-2 SNPs R21X, 4925G>A and 4733G>A and about the splice donor SNP rs41272114.

**Rs1853021** [101] (also known as +93C/T or c.-49T>C [7]; 5’ UTR SNP) is one of the earliest examples of linkage disequilibrium (LD) between a functional SNP and an isoform range. The T allele (which is the minor allele in the population, but the reference base in the human genome) creates an alternative start codon and reduces reporter gene expression, respectively *LPA* translation by 30–60% [101,102]. Accordingly, an association with lower Lp(a) is readily observed in Africans, where the SNP occurs across the isoform range (Fig. 5B). On the contrary, no effect on Lp(a) concentrations is detectable in Whites, where the T-allele is preferentially associated with HMW isoforms (24–34 KIV; Fig. 5B) [51]. This markedly diminishes its impact on Lp(a) concentrations in Whites although it is mechanistically present (explained in Fig. 6A).

In contrast, the 5’UTR SNP **rs1800769** [103] (also: 21G>A or +121G/A [7]) has been linked to a 90% increased promoter activity [102]. However, at a first glance, its effect on Lp(a) concentrations appears contradictory. In some studies in Europeans and Mexican Americans, it was found to be associated with lower Lp(a) plasma concentrations [81,100], while an association with increased *allele-specific* Lp(a) plasma concentrations has been reported in Europeans and African Americans by others [87,88,100]. This perceived contradictions are caused again by the association of this SNP with very large isoforms in Whites but not in African Americans (>30 KIV in Whites but 24–30 KIV African Americans, Fig. 5B). This leads to a net negative effect in Whites [81,87,100]. Accordingly, its negative effect on Lp(a) becomes positive also in Whites if the analyses are adjusted for the apo(a) isoforms [24,87,88,100]. This suggests that rs1800769, while being associated with lower overall Lp(a), may be associated with higher-than-expected Lp(a) in HMW isoforms (Fig. 6B). Unfortunately, the direct investigation of these two interesting regulatory SNPs in contemporary sufficiently powered studies is hampered by the fact that the former was not contained in the 1000 Genomes (1000G) imputation panel [7], while the latter is not contained in the Haplotype Reference Consortium imputation panel [104].

On the other hand, the LD of SNPs with isoforms can also be leveraged to ease Lp(a) research. Two SNPs have gained considerable attention (**rs10455872** [39] and **rs3798220** [39,90]) as they have been reported to tag LMW isoforms [39] and are thus used to circumvent laborious Western blotting in large studies [105]. Despite being very useful at population scale, it is important to note that this correlation is far from perfect at individual level. In a large study with ≈6000 individuals, only about half of the individuals with LMW apo(a) isoforms carried also one of these SNPs [105].

## 8 Allelic association between SNPs

Two recent examples illustrated how SNP-SNP LD structures in *LPA* and, more specifically, sole reliance on R^2^ as LD measure can be misleading. Fig. 8 summarizes the basic mechanism behind these confounding observations. **KIV-2 R21X** is a low frequency nonsense SNP in KIV-2 (MAF≈2%) [63,96]. In a study in ≈11,000 individuals, we found for this variant ≈12 mg/dL lower Lp(a) concentrations and a preferential association with HMW isoforms [96]. However, R21X did not provide additional information beyond the genotype of the *LPA* splice site mutation rs41272114 (discussed in next section). Indeed, we and others found that R21X occurs nearly exclusively on haplotypes that carry also the SNP rs41272114 [24,96]. This latter SNP is considerably more frequent (MAF ≈5%), which creates a misleading low R^2^ value with R21X. Together with the obvious functional consequence, this could have easily mislead researchers into assuming an independent function of R21X. Intriguingly, also a second very rare frameshift mutation in the KIV-2 has been observed recently on rs41272114-haplotypes [24].

Similarly, two methylome-wide studies on Lp(a) independently identified a rare *LPA* promoter SNP (rs76735376) [106,107] with a strong effect on *LPA* expression and Lp(a) concentrations (beta estimate +37 mg/dL [106]/+113 nmol/L [107]). The SNPs was restricted to isoforms 18–21 KIV repeats but R^2^ with the more frequent rs10455872 was <0.2 [106]. The adjustment for rs10455872 and isoforms cut the effect by 8-fold to +5.38 mg/dL [106]. Here the situation is very similar as for rs41272114 and KIV-2 R21X: rs76735376 is located nearly exclusively on rs10455872-haplotypes but the large MAF difference (1% vs 9%) induce a misleadingly low R^2^ value (Fig. 8).

Finally, a further layer of complexity is added by the fact that some rare functional SNPs may create subgroups within carriers of a more frequent SNP. These subgroups can present markedly different Lp(a) phenotypes than the parental haplotype. For example, rs10455872 is largely used as proxy for high Lp(a) but ≈5% of all carriers present low Lp(a) (<8.6 nmol/L). Said and colleagues identified a rare missense variant (**rs41267813**) in some rs10455872 carriers [108]. This SNP lowers the median Lp(a) concentrations in individuals with both SNPs to as little as 7% of the reference group (rs10455872 only), explaining the rs10455872 carriers with the surprisingly low Lp(a) concentrations [108].

## 9 SNPs causing null alleles

Up to 30–50% of the population express only one isoform at detectable levels despite being heterozygous at DNA levels [109]. Of course, this depends also on the sensitivity of the electrophoresis protocol since the amount of plasma applied to the SDS agarose gel depends on the Lp(a) concentration measured in plasma: in case of a high Lp(a) concentration with one major band responsible for the majority of Lp(a) in plasma, the second isoform might not be visible when the relative amount of the Lp(a) of this isoform applied to the gel falls under the detection limit. However, for the majority of probands with “null alleles” [14], two major mechanisms have been identified. On the one hand, large isoforms may fail to mature properly in the endoplasmatic reticulum and are degraded before being secreted [27,28,110]. On the other hand, LOF variants can suppress mRNA or protein production [24,63,111–113]. In contrast to several other examples described in this review, such variants may act independently from the background apo (a) isoform; however, depending on the apo(a) isoform with which they occur, the size of the Lp(a) lowering effect might be variable (Fig. 6B). The splice site mutation **rs41272114** [113] is the most frequent null allele mutation in Caucasians (MAF ≈3%) and explains ≈25% of all null alleles [113]. It has been largely used as genetic instrument for Mendelian randomization studies to support causal associations between Lp(a) concentrations and multiple outcomes [45,46,114], despite its effect on Lp(a) is overall rather moderate (–5 to –17 mg/dL). This is due to its preferential association with HMW isoforms (like KIV-2 R21X) [96]. Generally, splice defects appear to be rather frequent in *LPA.* At least five different null allele SNPs that abolish splice sites [24,64,113, 115] have been described, as well as three splice modifier SNPs that lower Lp(a) by 80–90% [24,29,30] (discussed in the next section) (Table 1 and Fig. 3).

Because of the cysteine-rich structure of the kringle domains, Mooser et al. proposed, already in the mid 1990s, that apo(a) might be particularly susceptible to missense mutations that impair secretion by preventing correct folding [83]. However, no such examples were known until Morgan et al. [112] recently showed that both **rs41259144** (p. Arg990Gln) in KIV-4 and **rs139145675** (p.Arg1771Cys) in KV impair apo(a) secretion by preventing correct folding. More such SNPs have been proposed by others also in KIV-2, KIV-6 and KIV-9 [24] and the same mechanism has been assumed also for two in-frame deletions in baboons (protease domain) [111] and humans (KIV-2) [30] (Table 1).

Taken together, these examples suggests that null alleles might be collectively quite common and may occur throughout the apo(a) protein (Table 1). While SNPs in canonical splice sites are easy to spot, missense variants causing null alleles are harder to identify in-silico. A thoughtful screening approach has been proposed by Morgan et al. [112], who prioritized *LPA* variants that cause plasminogen deficiency if occurring at homologous positions in plasminogen [112]. Since phylogenetic approaches for variant effect prediction are poorly applicable to *LPA* due to the fact that *LPA* is missing in most species, the rationale of Morgan et al. might be a useful approach for further endeavors.

Moreover, *LPA* SNPs may cause null alleles also without being clear-cut LOF mutations by simply lowering Lp(a) concentration below the assay detection limit if occurring on an allele with already low basal expression [29,30] (“operational null alleles” [14]).

## 10 SNPs in the KIV-2 region

KIV-2 can encompass the majority of the *LPA* coding region [29]. However, KIV-2 SNPs are not annotated in current SNP reference datasets like GnomAD [116] or TOPMed [117] because sequencing reads do not map uniquely, and the signal of genuine variants is diluted by reads from other KIV-2 units. An approach termed ‘batch sequencing’ [62,64,118] has been devised to circumvent the mapping issues and is illustrated in Supplemental Fig. 3. It makes use of the homology between the KIV-2 units to amplify and sequence all repeats as amplicon mixture, align all data to one KIV-2 and detect SNPs alike somatic mutations. Since many of these SNPs are present only in one or few KIV-2 repeats out of up to 80 repeats, this results in a condition that resembles somatic mutations with one or a few KIV-2 repeats carrying the mutation mixed into a vast majority of repeats that do not carry the mutation. Its early practical application was hampered by the limited sensitivity of Sanger sequencing [64,119], but ultra-deep next generation sequencing now provides sufficient sensitivity to one mutant KIV-2 in up to 80 KIV-2 repeats. Although technically challenging, this method opened new avenues to study this otherwise almost inaccessible region (see Refs. [24,38,57,64]). The first NGS batch sequencing study readily identified >500 KIV-2 SNPs in 123 individuals, including multiple missense, splice site and nonsense variants that were hiding in plain sight [57].

The two splicing mutations **KIV-2 SNPs 4925G>A** [29] **and 4733G>A** [30] discovered by this approach stand out as they explain 5% and 10% of isoform-adjusted Lp(a) variance [30]. In terms of variance explained, they thus represent the two most important genetic modifiers of Lp(a) concentrations besides the apo(a) isoform size. Indeed, they are remarkable prime examples that recapitulate many complexities of the genetics of *LPA.* Both SNPs are very frequent with MAFs of 13% and 22%, meaning that they are found in ≈22% and ≈38% of the European population, respectively. Both show widely varying MAF differences between various ancestries [29,30]. 4925G>A is found mostly in isoforms at the boundary between LMW and HMW isoforms (≈19–25 KIV) (Fig. 2D). KIV-2 4925G>A decreases Lp(a) by ≈ 30 mg/dL in individuals with LMW apo(a) isoforms explaining ≈19% of isoform-adjusted Lp(a) variance; it decreases Lp(a) by ≈10 mg/dL in individuals with only HMW isoforms explaining ≈1.6% of isoform-adjusted Lp(a) variance. This variant also partially accounts for the astonishing drop in median Lp(a) concentrations at 23–25 KIV repeats (Fig. 2D). In a large German cohort, the median Lp(a) concentration of the 23 KIV isoform group increased from ≈10 to ≈23 mg/dL when 4925G>A carriers were excluded, which underscores the pronounced Lp(a)-lowering effect of this variant [29]. Despite these strong effects on Lp(a) concentrations, its effect on Lp(a) variance at population scale is detectable only in isoform-adjusted regression models (R^2^ = 0.2% not adjusted vs. R^2^ = 6.1% isoform-adjusted) [29]. This phenomenon is even more notable in the HMW range (R^2^ = 0.02% vs. 1.6%) [29] and is caused by the fact that within the HMW isoform range the SNP occurs on rather short HMW isoforms. In turn, these present relatively high Lp(a) and 4925G>A reduces their Lp(a) concentrations to a value that is close to the median of the overall HMW group. Therefore, the effect is not visible if the isoform background is not considered (see Fig. 6C).

**Fig. 7:**
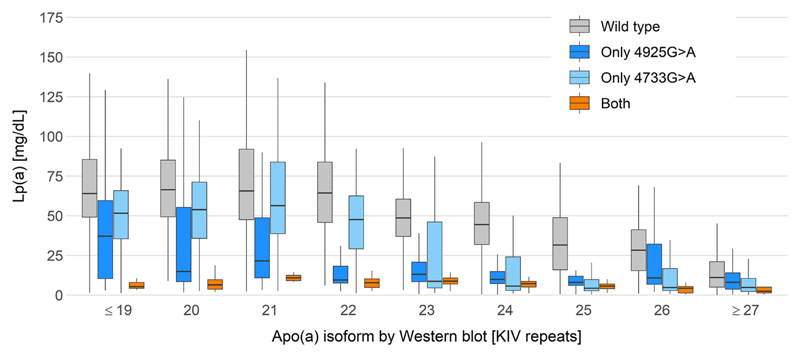
Effect of KIV-2 SNPs 4925G>A and 4733G>A on Lp(a). Compound heterozygosity with KIV-2 SNPs 4925G>A and 4733G>A lowers Lp(a) by 32 mg/dL and virtually abolishes Lp(a) variance over the whole isoform range, resulting in a nine-fold narrower interquartile range in carriers than in wild type individuals (4.6 vs. 42.1 mg/dL). Data is from Fig. 4B of Schachtl-Riess et al., 2021 [30]. Outliers omitted for better representation. Where necessary, isoforms are grouped to encompass at least five individuals per group.

**Fig. 8:**
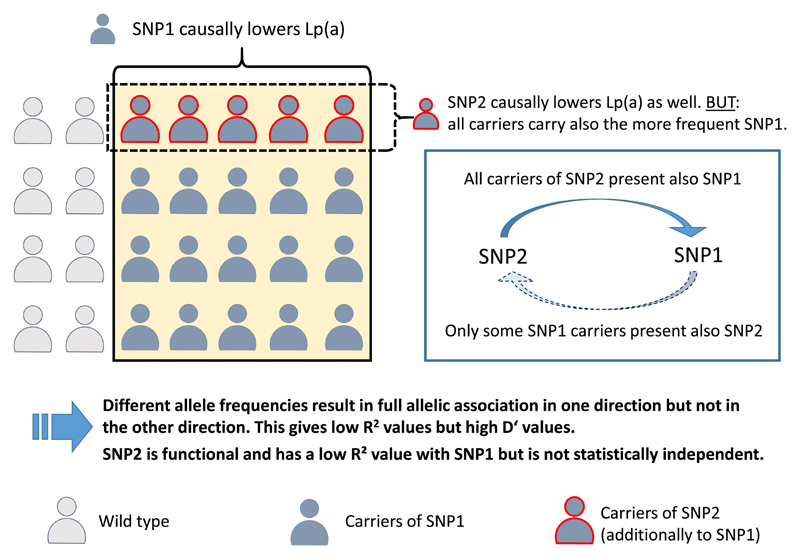
Example of how allelic association between a frequent and a rarer functional SNP might mislead association studies. The functional *LPA* SNP2 occurs on the same haplotype as the second functional SNP1, which is, however, considerably more frequent. Due to the different MAFs, the R^2^ value between these two SNPs will be low and the SNPs might be easily regarded as independent (albeit D′ will be high). SNP2 alone will show an association with Lp(a), but this association will vanish if also SNP1 is included in the regression model. SNP2 is not statistically independent and adds little or nothing to the genetic variance explained by SNP1. Two such examples are described in section 8. “Allelic association between SNP” (SNP pairs rs41272114/KIV-2 R21X and rs76735376/rs10455872).

The second KIV-2 splicing SNP 4733G>A is associated with a more moderate Lp(a) reduction of –13 mg/dL when adjusted for apo(a) isoforms. However, it is very frequent in Whites and it is found across the whole isoform range with some preferential association to isoforms 24–33 KIV. Its high frequency makes it a major determinant of discordant Lp(a) phenotypes and the most important genetic factor affecting Lp(a) variance in Whites after the apo(a) isoforms [30]. The moderate but lifelong Lp(a) reduction translates into an 9% lower hazard ratio for CAD [30]. Mechanistically, it induces a splicing defect causing an in-frame deletion, which removes a structure-bearing cysteine residue [30] and likely induces an secretion defect caused erroneous folding (alike rs41259144 and rs139145675 [112]). In a German population, compound heterozygosity with KIV-2 4925G>A (about 5% of the population) is associated with –32 mg/dL lower median Lp(a) and, as both alleles are blunted, almost no Lp(a) variance (Fig. 7) [30].

As both SNPs efficiently dissociate Lp(a) concentrations from isoform size (Fig. 1D), they might be interesting genetic tools to better dissect the interplay of Lp(a) concentrations and isoform size. Accordingly, a seminal study in >140,000 Icelanders, has recently used KIV-2 4925G>A to investigate whether LMW isoforms present an independent atherogenic potential [38]. In line with our earlier study [29], the authors found that the atherogenic potential is conferred through the Lp(a) concentration meaning that subjects with LMW isoforms but low Lp(a) concentrations (caused e.g. by the 4925G>A variant) were not at an increased CAD risk [38]. As discussed in another review of this series [120], this demonstrates that the Lp(a) concentration has a stronger information content than certain SNPs since it comprises the entire genetic information as well as non-genetic (environmental) factors.

## 11 Differences in mutation patterns between ancestries

Ancestry is a major modifier of Lp(a) concentrations [19,21,22] and a two-fold variation is observed even within Europe [65,85,121]. These differences extend also to MAFs, SNP haplotypes and association with isoform ranges (Table 1, Figs. 4 and 5B). For example, the LOF variant **rs41272114** is twice as frequent in Admixed Americans than in Europeans (8% vs. 3% MAF) and a MAF of even 18% was observed in a small sample of 85 Peruvians from the 1000G project [96]. Conversely, the splice site mutation **rs143431368** is very rare globally (≪1% MAF) but frequent in Finns (MAF≈5%) [115]. The high impact KIV-2 SNPs discussed before range from to 0–22% MAF globally [29,30]. Also the LMW isoform-tagging SNP **rs3798220** presents a particularly pronounced heterogeneity across ancestries. It is absent in Africans, rare in Europeans (MAF≈2%), moderately frequent in South Asians (MAF = 12%) and very frequent in Hispanics (up to 42% MAF) [22,39,122,123]. However, it does not tag LMW isoforms in Asians [123] and Hispanics [22]. In Europeans, it is also in partial LD with the strong GWAS hit **rs140570886** (associated with +43 mg/dL higher isoform-adjusted Lp(a) [77]; Table 1), which in turn presents nine-fold higher MAF in Admixed Americans and Latinos than in all other continental groups of GnomAD.

Many more such examples exist and leveraging these differences in comparative cross-ancestry genetic studies might help pinpoint functional SNPs. However, it needs to be considered that SNPs may segregate with very different isoform ranges between populations (Fig. 6B and Table 1). As already pointed out by Utermann [124], this can mislead fine-mapping efforts. In cross-ancestry studies, effect heterogeneity between ancestries is often interpreted as a sign that a GWAS hit is actually rather a proxy SNP than a causal SNP [124]. However, as discussed above, even genuine functional SNPs may present considerably different effects if occurring in different isoform ranges across populations. While intensifying genetic studies in non-Whites will definitely be fruitful, proper care is required. Isoform data is still rare, especially for large non-White populations. Considerable efforts will thus be necessary to map the association between SNPs alleles and apo(a) isoforms across ancestries on a large scale and in a standardized manner. Western blotting is very laborious, which has precluded such endeavors until now. Recent advances in haplotype phasing and imputation algorithms are, however, opening new avenues to approach this shortcoming [24,38].

## 12 Findings from recent genomic studies

Many candidate gene, sequencing and GWAS studies have searched for genetic variants that modulate Lp(a) concentrations [77,108, 125–132]. Notwithstanding the many entanglements discussed above, these studies have been very successful and have identified dozens of independent SNPs (tagging >2000 significant variants) in a ≈2 megabases region around *LPA* [77,108,133]. These SNPs have been recently used to construct effective genetic risk scores that explain up to ≈70% of Lp(a) concentration variance [43,44,133,134]. They represent valid genetic surrogates for direct Lp(a) quantification, with similar distribution and similar association to cardiovascular outcomes [43,44,134]. While for the time being a direct Lp(a) quantification is cheap and easy, these scores may become efficient screening tools in the future as the availability of genomic data in clinical care is constantly increasing.

Nevertheless, still some gap exists to reach the ≈90% variance that is explained by the complete *LPA* locus. Further informative variants might be masked by complex associations with isoforms or non-additive epistatic effects. So far, only one isoform-adjusted GWAS (n > 13,000) has been performed [77] but, interestingly, the isoform-adjusted regression model still detected 30 independent hits representing 1961 SNPs in the *LPA* gene locus [77]. These SNPs might tag further functional SNPs that create discordant phenotypes (e.g. the GWAS hit rs75692336 is a proxy SNP for KIV-2 4925G>A). However, disentangling functional SNPs from simple isoform-tagging SNPs will be a major task as these two roles are not mutually exclusive (as exemplified by KIV-2 4925G>A [29]). They are also modified by the isoform sizes and by non-linear epistatic effects. These are not captured well by standard regression models [24,104]. For example, Zeng et al. found that the effect of **rs140570886** on Lp(a) and CAD depends on the haplotype of **rs1800769 (promoter)** and **rs9458001 (enhancer)**, while, vice versa, only the minor allele of rs140570886 enables a strong effect of rs1800769 on Lp(a) [104].

Despite the value of apo(a) isoform information in Lp(a) studies, apo (a) Western blotting is not feasible at very large scale. This limits the scale of in-depth studies. Two very recent studies have provided a major leap forward to address this issue [24,38]. Both studies estimated the total KIV-2 number at DNA level by using the NGS coverage and apportioned it to the two parental haplotypes using advanced phasing procedures [24,38]. This gave a “diploid KIV-2 content” representing an imputed KIV-2 genotype (iKIV-2). In Mukamel et al. [24], this iKIV-2 explains 61% of Lp(a) variance in the UK Biobank [135], which is remarkably close to the upper bound of variance explained by isoforms measured directly by Western blotting (30–70% [1]). This is even more impressive when considering that it does not take into account non-expressed alleles. When comparing the iKIV-2 from whole exome sequencing data to direct KIV-2 sizing using optical mapping [136] (a method similar to Fiber-FISH [137]), the authors observed a good correlation (R^2^ = 0.67).

The authors subsequently used the phasing algorithm to assign all *LPA* SNPs to the background iKIV-2 allele and finally restricted the analyses to 24,969 heterozygous null allele carriers [24]. This removed the confounding effect of the second allele and produced a large haploid Lp(a) dataset. While this concept had been proposed earlier [80,81], only current data from the UK Biobank allows to apply it at large scale. Using stepwise conditional analysis the authors finally identified 23 very promising *LPA* sequence variants with MAFs from 0.01% to 28% that likely causally affect Lp(a) production by the respective allele. These include known null alleles, the functional KIV-2 SNPs 4925G>A [29], 4733G>A [30] and R21X [63,96], and the regulatory SNPs rs1853021 [102] and rs1800769 [103]. When accounting also for cis-epistatic and non-linear effects, these SNPs raised the explained Lp(a) variance to an impressive 83%, respectively 90% of heritable variance) [24]. 43% of all European haplotypes presented at least one modulator SNP but only 13% of the African alleles [24]. The frequency differences in these 23 SNPs largely explained the cross-ancestry differences in Lp(a) [24]. Provided replication of these findings and thorough validation against directly measured isoforms, the wide implementation of these algorithms promise unprecedented opportunities to scale up isoform-adjusted analyses and possibly even accurately predict Lp(a) from genetic data. Unfortunately, to date these algorithms have not been distributed as widely applicable bioinformatic tool.

Given the strong impact of *LPA,* other major regulators have been elusive for a long time. An exception is the APOE2 allele which lowers Lp(a) markedly by 3 mg/dL per copy [77,129]. A GWAS in the UK Biobank finally identified 37 additional loci [108], but their effects were mostly very small (<5% of the top hit *LPA* rs10455872). Most intriguingly, among the many candidate receptors for Lp(a) that had been proposed (reviewed in Ref. [138] and in this series [139]), only a minor effect of *LDLR* (≈2% of the effect of the top hit rs10455872) has been identified by those GWAS, while a previously reported association of *SCARB1* variants with high Lp(a) [140] was not replicated. On the other hand the effect size in GWAS may not necessarily reflect the physiological relevance of a gene [141]. Therefore, it will be interesting to see whether these associations will still shed new light on the machinery involved in the metabolism and catabolism of Lp(a). The detection of genes that have been described before (*LDLR* [138], *APOH* [142]) or are known from pharmacological intervention (*CETP* [143], *PCSK9* [144], *LDLR* [144]) may be promising. However, it will not be straightforward to disentangle genes which show up in GWAS for both phenotypes, LDL cholesterol and Lp(a), since the cholesterol content of Lp(a) is also included in the LDL cholesterol measurement.

## 13 Outlook

Advances in genomic technologies, bioinformatics and statistical genetics have generated considerable insights into how SNPs regulate the Lp(a) concentrations beyond the apo(a) isoforms. What do these complex genetic studies tell about Lp(a) biology? The intrinsic biological role of Lp(a) is still unknown. It acts as a preferential carrier of OxPL (reviewed in Ref. [5] and in this series [6]), but this could be rather an acquired function than its native role (given the large number of people with very low to even null Lp(a)). The GnomAD dataset [116] with >120,000 exomes reports a very high mutational burden for *LPA,* with an LOF ratio (observed LOF number/expected LOF number under neutrality [116]) of even 1.3. It is not clear whether and how this can be reconciled with a critical biological function, which has recently led to the intriguing speculation that *LPA* might indeed be a large translated pseudogene lacking an intrinsic physiological function [145]. While definitely provocative, this may fit to the genetic data and such a rationale may have important implications on the direction of further Lp(a) research, e.g. the search for specific receptors. On the other hand, given the existence of >15,000 pseudogenes in the human genome, a pseudogene with such a pronounced pathophysiological function and being translated to a large protein might be very unusual.

It should be pointed out that the majority of genetic data on *LPA* available to date has been generated in Caucasians. It is unknown whether the LOF frequency is similarly high also in other populations, especially in Africans, which present higher median Lp(a). If not, it would be interesting to interrogate whether this reflects an unknown selective pressure. If yes, it would be intriguing to get to know what other factors are counterbalancing those LOFs.

Indeed, most SNPs, for which a causal mechanism has been identified, lower Lp(a), with at least ten clear-cut LOF mutations identified so far and many more postulated (Table 1 and references [24,29,63,101, 113,115]). Every isoform group presents many individuals with Lp(a) that is considerably lower than the group median Lp(a), but a similar amount of people is seen when the isoform-associated Lp(a) deviates at least as much towards high Lp(a). Some SNPs that raise Lp(a) have been found (mostly by association studies), but none present a similarly large effect as the KIV-2 SNPs 4733G>A and 4925G>A. Only for rs1800769 some direct functional impact on high Lp(a) concentrations has been shown. It is fully unclear whether the genetic architecture of high Lp(a) is similar to that of low Lp(a). The complex genetics of Lp(a) may still hide some surprises.

## Supplementary Material

Supplementary File

## Figures and Tables

**Table 1:** Genetic variants of interest in the *LPA* gene region. This table summarizes information about selected variants that have been either extensively discussed in the literature or that present notable functional effects. Importantly, the table does not report all reported *LPA* SNPs as many more variants have been mentioned in publications without further discussion and can be found in the references cited in the table, in Refs. [7,23,75,92,128,148,149], in recent fine mapping efforts [24,108,130] and in GWAS studies [39,108,126,128,129,131,150–152]. GWAS have recently identified also some loci outside *LPA,* but with mostly small effects [77,108,129, 131]. The minor alleles of variants outside the KIV-2 region are according to the gnomAD 2.1.1 exome dataset for coding SNPs (n = 125,748 exomes and 15,708 genomes) and the gnomAD 3.1.2 whole genome dataset for non-coding SNPs (n = 76,156 genomes). Due to space limitations and because nearly all studies have been done in individuals of White European ancestry, MAF is given only for the Non-Finnish Europeans group. Fig. 4 shows the MAFs of selected SNPs in other major continental groups of gnomAD. Alleles and MAFs of variants within the KIV-2 are from the respective publications. Effects reported from GWAS are beta estimates from regression models. Effect on protein is annotated according to NP_005568.2. Unless indicated differently, isoform ranges in the table are from Caucasian samples, as little data is available for Non-Caucasians.

Gene region	rsID (Ref>Alt), effect	Alternative names	MAF_NFE_	Reported effects	Isoform range^a^	References
Enh.	rs186696265 (C>T)		0.0149	Reported by multiple GWAS on Lp (a), lipids and cardiovascular endpoints. Associated with Lp(a) changes of +64 mg/dL (SNP alone), +49 mg/dL (isoform-adjusted) and +24.75 mg/dL (adjusted for isoforms and other GWAS hits), respectively. OR for CAD 1.73 in CARDIoGRAM-plusC4D consortium. Partial correlation with rs3798220.	NR	[77,78]
Enh.	rs7760010 (C>A)	−1712G>T	0.004	Decreases reporter gene activity by 40%. Associated with 40% lower Lp(a) from the mutant allele.	NR	[76]
Enh.	rs7758766 (G>T)	−1617C>A	0.163	Decreases reporter gene activity by 30%. Detected in GWAS only after isoform-adjustment.	NR	[76,77]
Enh.	rs9347440 (C>T)	−1230A>G	0.533	Increases reporter gene activity by 250%. Associated with 70% higher Lp(a) derived from the mutant allele.	<24 KIV	[76]
Promoter	rs76735376 (C>T)		0.0127	Located in a CpG site identified by methylome-wide association analysis. Associated with +37 mg/dL/+114 nmol/L Lp(a) (+20 mg/dL after isoform adjustment) but in a joint model most signal is absorbed by rs10455872. Independent effect is ≈+5.4 mg/dL.	≈19-20 KIV	[106,107]
Promoter	STR at hg38, chr6:160,665,587-160,665,631 (≈6-12 repeats)	Pentanucleotide repeat, PNR, TTTTA repeat, TAAAA repeat	NA	No causal effect on *LPA* expression but alleles show association with various isoform ranges. Alleles PNR10 and PNR11 tag discordant phenotypes (PNR11: LMW with Lp(a) < 3 mg/dL; PNR10 tags different subgroups, one being a discordant phenotype with < 24 KIV, but Lp (a) < ≈15 mg/dL).	PNR8: 15–40 KIV; PNR9: 25–37 KIV; PNR10: 26–35 KIV and 19–23 KIV depending on specific haplotypes.; PNR11: 18-23	[68,83,101, 153–155]
Promoter	rs1800589 (T>C)	−914G>A, -772G>A^b^	0.553	Effect on *LPA* transcription was proposed, but functional studies did not substantiate this. T allele reported to be in LD with rs1853021-A and rs1800769-A.	NR	[88,101,153,154]
5’ UTR	rs1853021 (A>G)	+93 C/T, -49T>C^c^	0.857	T allele introduces an alternative translation start codon and reduces reporter activity and protein production by 60%. ≈10 mg/dL lower Lp(a) in Africans. Effect is masked in Whites due to association with moderately large isoforms.	∞24–34 KIV in Caucasians in Ref. [51]; >26 KIV in Ref. [87]; Broad range in Africans	[51,87,101–103]
5’ UTR	rs1800769 (C>T)	+121 G/A, -21G>A,^c^	0.168	Increases promoter activity. Increases Lp(a) by 40–60%; common in Africans. Proposed to modulate the effects of the GWAS hit rs140570886 via epistatic interactions with rs9458001.	NFE: >32 KIV in Ref. [87]; ≈30–36 KIV in Ref. [100] and in Ref. [24] e; AFR: 24–30 KIV [100], ≈23–32 in [24]; Mexicans: large isoforms >780 kDa [81]	[24,81,87,100,102–104]
5’ UTR	rs1623955 (T>G)		0.00021	Very rare putative regulatory variant causing null alleles via an unknown mechanism.	NR	[24]
KIV-2	No rsID (C>T) KIV-2 p.Arg21Ter nonsense	KIV-2 p.R21X p. Arg20Ter^d^	≈0.0078 to 0.02	Nonsense mutation in KIV-2 causing null alleles. Most gene alleles carrying p.Arg21Ter carry also rs41272114. Associated with –9.9 and –12.5 mg/dL in two general populations.	27–32 KIV	[24,63,96]
KIV-2	No rsID (A>AGCTT) KIV-2 Trp36fs		0.0016	Frameshift variant causing null alleles. Most gene alleles carrying this variant carry also rs41272114 on the same allele.	NR	[24]
KIV-2	No rsID (A>C) KIV-2 p.Tyr51Asp	KIV-2.1 Y51D	0.0033	Missense variant causing null alleles.	≈27–30 KIV^e^	[24,57]
KIV-2	No rsID (C>T) Splice site	KIV-2.1 +0C>T	0.0001	Splice site variant causing null alleles.	NR	[24]
KIV-2	No rsID (G>A) Splice site	KIV-2.1 +1G>A	0.0053	Rare splice site variant causing null alleles.	≈20–30 KIV^e^	[24,64]
KIV-2	No rsID (C>T) Splicing modifier	4733G>A KIV-2.2 -11G>A	≈0.22	Strongest genetic contributor to Lp(a) variance in Caucasians after the smaller isoform. Compound heterozygosity with 4925G>A reduces Lp(a) by 31.8 mg/dL and narrows the interquartile range by nine-fold (42.1–4.6 mg/dL) compared to the wild type.	Whole isoform range, but preponderance in ≈24–33 KIV	[24,30,57]
KIV-2	No rsID (C>T) Splicing modifier	4925G>A, G4925A, KIV-2.2 +0G>A	≈0.13	MAF≈13% in NFE. Reduces Lp(a) by 31 mg/dL in LMW isoforms; explains 19% of isoform-adjusted Lp(a) variance. Second strongest genetic contributor to Lp(a) variance after LMW isoforms and KIV-2 4733G>A. Very pronounced differences between populations, ranging from 0 to 27% carriers in the population.	19–25 KIV	[24,29]
KIV-3	rs75692336 (C>A) intronic		0.135	Tagging SNP for KIV-2 +4925G>A (r2 = 0.82, D’ = 0.99). Associated with — 9.67 mg/dL in an isoform-adjusted GWAS (detectable only in the isoform-adjusted model).	19–25 KIV	[29,77]
KIV-4	rs41259144 (C>T) p.Arg990Gln		0.019	Missense variant causing null alleles due to impaired protein folding and secretion. —14 mg/dL in a GWAS (—7 in a joint model with all other GWAS hits).	≈34–37 KIV^e^	[24,77,112]
KIV-5	rs41270998 (A>G), Splicing modifier		0.0046	Very rare SNP in the polypyrimidine tract 6 bp downstream of the first exon of the KIV-5. Likely disrupts splicing. Associated with reduced allelic Lp (a) expression.	≈ 14–24 KIV^e^	[24]
KIV-6	rs140570886 (T>C), intronic		0.0153	Associated with strongly increased Lp(a) (+43 mg/dL isoform-adjusted Lp(a), +23.78 mg/dL joint analysis with other GWAS hits). OR 1.46–1.77 for CAD. Rs9458001 (enhancer) and rs1800769 (promoter) SNPs exert an effect on Lp(a) and CAD only in haplotypes with rs140570886-T allele. Better predictor for Lp(a) concentrations than rs3798220.	≈19–25^e^	[38,77,104]
KIV-6	rs201297680 (A>T) p.Cys122Ser		0.00015	Putative very rare null allele variants.	NR	[24]
KIV-7	rs10455872 (A>G), intronic		0.069	Strongest GWAS hit in Lp(a) (p < 10e-20,000). Explains about 25% of Lp(a) variance by partially tagging small isoforms. Associated with ≈ +30 mg/dL Lp (a). About half of all LMW isoform carriers carry also this SNP. Frequent only in Caucasians. Rare in Africans (MAF <1%). Associated with increased *LPA* expression in liver samples.	Africans: 16–17 (but SNP is rare) [22] NFE: 16–18 [22], 17–20 [39], 17–22 [106] Hispanics: 16–17 [22]	[22,39,106, 108,109, 131]
KIV-8	rs41272110 (T>G), p.Thr1399Pro	p.Thr3888Pro KIV-8 Thr12Pro KIV-8 Thr23Pro	0.141	Frequent polymorphism. Effect on Lp(a) is controversial. Some early studies found an Lp(a)-reducing effect after adjusting or stratifying by isoform. Was detected in GWAS only after isoform-adjustment.	NFE: 23–29 [87], 21–25 [94] AA: 18–25 [22] Hispanics: 19–26 [22]	[22,87,94,100]
KIV-8	rs76144756 (G>A), p.Pro1428Leu		0.006	Rare variant associated with reduced allelic Lp(a) expression.	NR	[24]
KIV-8	rs41272114 (C>T) Splice site	+1 G>A G+1inKIV-8A, G+1/inKIV-8A	0.039	Most frequent LOF-mutation in Caucasians, accounting for ≈25% of all null alleles. Associated with reduced Lp(a) (–0.62 SD in Emdin et al.; –5 mg/dL in Mack et al. [77] and Kyriakou et al. [45]). About 0.12–0.21 OR reduction for CAD/CHD. Frequencies range from ≈0% to 18% between populations.	≈27–33 in Ref. [96] (n = 12 by pulsed-field gel electrophoresis) No clear association in Ref. [24] ^e^	[23,24,38,45,46,77,94,96,100,113]
KIV-9	rs41267813 (G>A) p.His1534Tyr		0.0014	Found in haplotypes carrying rs10455872, reduces Lp(a) by 13-fold, causing small isoforms with low Lp(a).	19–21 KIV in Ref. [24] ^e^ Not reported in Ref. [108], but likely similar to rs10455872.	[24,108]
KIV-9	rs6938647 (A>C) Intronic		0.779	Tagging SNP for KIV-2 +4733G>A.	Whole isoform range, but the A-allele is more frequent in ≈23–30 KIV.	[30]
KIV-10	rs143431368 (T>C), Splice site		0.003	Splice site mutation. Ten times more frequent in Finns (MAF≈5%) than in Non-Finnish Europeans.	27–31 KIV [24]	[24,115]
KIV-10	rs1801693 (A>G) p.Met1679Thr	KIV-10 Met66Thr, p.Met4168Thr, Met/Thr KIV 37, Met/Thr KIV-10, NcoI polym. (alleles N+/N-; N+ being adenine)	0.688	Frequent missense variant with no effects on apo(a) function. No effect on Lp(a) concentrations in Caucasian, but positive association of the A allele with Lp (a) in African Americans and Hispanics.	AA: 20–26 (A/A genotype) [22] NFE: 18–29 [22], possibly with some preponderance of 26–33 KIV [88], but not confirmed [87] Hispanics: 19–28 (A allele); 25–34 (G allele) [22]	[22,87,88,94,156,157]
KIV-10	rs1211014575 (A>G) p.Trp1685Arg	KIV-10 Trp72Arg	NA^f^	Very rare SNP located in the lysine-binding pocket of KIV-10. Abolishes lysine and oxPL binding capacity of KIV-10. It has been speculated that it might produce Lp(a) particles that are “benign” from the cardiovascular point of view, but its very low frequency (gnomAD: 3e10^−5^) has prevented direct testing of this hypothesis.	NR	[5,97,158]
KIV-10	rs41267811 (C>G), p.Ser1694Ter		0.00022	Very rare nonsense mutation causing null alleles.	NR	[24]
KV	rs139145675 (G>A) p. Arg1771Gln		0.0013	Missense variant causing null alleles due to impaired protein folding and secretion.	≈ 19–25 KIV^e^	[24,112]
Protease domain	rs3798220 (T>C) p.Ile1897Met	p.Ile4399Met, I4399 M	0.017	Rare variant associated with small isoforms, particularly high Lp(a), increased mRNA expression in GTEx and higher oxPL load. Is associated with ≈+45 mg/dL and explains ≈8% of Lp(a) variance. Increased clot lysis time and decreased clot permeability in Caucasian and methionine allele triggers amino acid oxidation. No differences in plasminogen inhibition capacity or hepatocyte apo(a) secretion rate.	AA: 21–33 [22] NFE: 17–19 [22] NFE: 19–21 [39] Hispanic: 24–34 [22] Asians: 32 (mean) [123]	[22,39,90,123,159–161]
Protease domain	rs41267809 (A>G), p.Leu1961Pro		0.023	Missense variant associated with 93% lower allelic Lp(a) expression. Associated with – 6.8 mg/dL in a GWAS.	No clear association	[24,77]
Protease domain	rs201306475 (C>T), Splice site		0.00033	Splice site SNP causing null alleles.	NR	[24]
Protease domain	rs3124784 (C>T) Arg2016Cys		0.284	Frequent variant associated with 28% lower allelic Lp(a) expression.	NR	[24]
Protease domain	rs41267807 (T>C) p.Tyr2023Cys		0.015	Missense variant associated with 89% lower allelic Lp(a) expression. Associated with –5 mg/dL in GWAS.	No clear association	[24,77]

rsID: dbSNP identifier. Ref: reference allele. Alt.: alternate allele. MAF: minor allele frequency. AA: African Americans. AFR: Africans. NFE: Non-Finnish Europeans. EUR: Europeans. Enh: enhancer region DH-III [73]. STR: short tandem repeat. Polym: polymorphism. HWE: Hardy-Weinberg equilibrium, NR: not reported.

a Unless otherwise indicated, the isoform association relates to the minor allele.

b Named SNP -772 in some papers [88,101,102] due to numbering starting from the transcriptional start used by Wade et al., 1994 [66].

c Numberings are from the transcription start and the translation start, respectively.

d Numbering of the amino acids in KIV-2 may vary by 1 between studies, depending on which amino acid is counted as first KIV-2 amino acid, because the first KIV-2 triplet starts still in the last KIV-1 exon.

e Estimate based on phasing the KIV-2 CN from sequencing data by using long-range SNP haplotypes to infer which individuals have inherited the same genomic segment, i.e. the same allele [24,38].

f Reported in GnomAD 2.1.1 only in one Finnish individual (MAF = 0.0003).

## Key points box 1

The genetics of Lp(a)

- Lp(a) is the lipoprotein with the strongest genetic regulation: >90% of the concentrations are genetically determined.
- A copy number variation within the *LPA* gene with 10 to >50 KIV repeats results in >40 apo(a) isoforms. A major fraction of the Lp(a) concentrations is explained by an inverse correlation between the number of KIV repeats and the Lp(a) concentrations.
- Discordant Lp(a) phenotypes are situations in which the mentioned inverse correlation is disrupted by other genetic variants. They are quite frequent and mostly caused by single nucleotide polymorphisms (SNPs) that lower Lp(a) concentrations. Very few SNPs are known that increase Lp(a) additionally to the isoforms.
- The *LPA* gene contains a very large number of different loss of function mutations over a wide range of allele frequencies, which concomitantly shape the Lp(a) trait.
- It is common that functional *LPA* SNPs occur only in defined isoform size ranges.
- The well-known SNPs rs10455872 and rs3798220 are usually observed with small apo(a) isoforms and are associated with high Lp(a) concentrations but do not tag these perfectly.
- The KIV type-2 region has become accessible for mutation detection only recently. This revealed a large number of new and partially functional SNPs.
- The splice site variants 4925G>A and 4733G>A in the KIV type-2 region are highly frequent (22% and 38% carriers, respectively) and result in a pronounced decrease in Lp(a) concentrations (≈30 and 13 mg/dL, respectively) resulting in a lower risk for cardiovascular disease.
- The allele frequencies of several SNPs differ considerably across ancestries, which partially explains the differences in Lp(a) concentrations between ancestries.

## Key points box 2

How can genetic research help to understand current mysteries of Lp(a)?

Many mysteries about Lp(a) remain unsolved and are discussed in multiple reviews of this series. For some, current genetic approaches can provide important insights about the direction to take in this journey (discussed in the section “Why are we keen to understand the genetic regulation of Lp(a)?”).

- **The link between diabetes and Lp(a).** The causal link between Lp(a) and cardiovascular diseases has been largely accepted but the relationship with diabetes is still unclear. Mendelian randomization studies have been tremendously successful in providing proof of causal links between phenotypes and outcomes, but for Lp(a) and diabetes we still miss the right genetic instrument.
- **Differences between ancestries.** Differences in the Lp(a) trait between ancestries might be caused by differences in the frequency and the isoform association of multiple *LPA* SNPs with large impact on Lp(a) concentrations and Lp(a) distribution. Yet, also environmental exposures and inflammatory burden have been proposed as causal factors. A comprehensive catalogue of the functional genetic variation in *LPA* across ancestries is urgently needed to define the true remainder, which may then be attributable to polygenic influences or environment.
- **The metabolism and receptors of Lp(a).** Given the nearly monogenic architecture of Lp(a), metabolic and whole-genome studies of individuals or pedigrees with notable phenotypes might provide novel insights. However, it is not straight-forward to identify “notable Lp(a) phenotypes” that are truly caused by metabolic differences because multiple, partially even frequent SNPs can uncouple the apo(a) isoform make-up from the Lp(a) concentrations. This generates “notable Lp(a) phenotypes” by purely genetic means. A full catalog of such variants will be necessary before being able to accurately pinpoint individuals whose notable Lp(a) phenotype can be safely attributed to polygenic effects or metabolic differences.

## References

[1] Kronenberg F, Utermann G (2013). Lipoprotein(a): resurrected by genetics. J Intern Med.

[2] Nordestgaard BG, Langsted A (2016). Lipoprotein(a) as a cause of cardiovascular disease: insights from epidemiology, genetics, and biology. J Lipid Res.

[3] Tsimikas S (2017). A test in context: lipoprotein(a): diagnosis, prognosis, controversies, and emerging therapies. J Am Coll Cardiol.

[4] Nordestgaard BG, Chapman MJ, Ray K, Boren J, Andreotti F (2010). Lipoprotein(a) as a cardiovascular risk factor: current status. Eur Heart J.

[5] Boffa MB, Koschinsky ML (2019). Oxidized phospholipids as a unifying theory for lipoprotein(a) and cardiovascular disease. Nat Rev Cardiol.

[6] Koschinsky ML, Boffa MB (2022). Oxidized phospholipid modification of lipoprotein (a): epidemiology, biochemistry and pathophysiology. Atherosclerosis.

[7] Schmidt K, Noureen A, Kronenberg F, Utermann G (2016). Structure, function, and genetics of lipoprotein (a). J Lipid Res.

[8] McLean JW, Tomlinson JE, Kuang WJ, Eaton DL, Chen EY (1987). cDNA sequence of human apolipoprotein(a) is homologous to plasminogen. Nature.

[9] Lawn RM, Schwartz K, Patthy L (1997). Convergent evolution of apolipoprotein(a) in primates and hedgehog. Proc Natl Acad Sci Unit States Am.

[10] Kraft HG, Köchl S, Menzel HJ, Sandholzer C, Utermann G (1992). The apolipoprotein (a) gene: a transcribed hypervariable locus controlling plasma lipoprotein (a) concentration. Hum Genet.

[11] Lackner C, Boerwinkle E, Leffert CC, Rahmig T, Hobbs HH (1991). Molecular basis of apolipoprotein (a) isoform size heterogeneity as revealed by pulsed-field gel electrophoresis. J Clin Invest.

[12] Marcovina SM, Zhang ZH, Gaur VP, Albers JJ (1993). Identification of 34 apolipoprotein(a) isoforms: differential expression of apolipoprotein(a) alleles between American blacks and whites. Biochem Biophys Res Commun.

[13] Utermann G (1989). The mysteries of lipoprotein(a). Science.

[14] Utermann G, Menzel HJ, Kraft HG, Duba HC, Kemmler HG (1987). Lp(a) glycoprotein phenotypes. Inheritance and relation to Lp(a)-lipoprotein concentrations in plasma. J Clin Invest.

[15] Koschinsky ML, Beisiegel U, Henne-Bruns D, Eaton DL, Lawn RM (1990). Apolipoprotein(a) size heterogeneity is related to variable number of repeat sequences in its mRNA. Biochemistry.

[16] Kraft HG, Lingenhel A, Bader G, Kostner GM, Utermann G (1996). The relative electrophoretic mobility of apo(a) isoforms depends on the gel system: proposal of a nomenclature for apo(a) phenotypes. Atherosclerosis.

[17] Boerwinkle E, Leffert CC, Lin J, Lackner C, Chiesa G (1992). Apolipoprotein(a) gene accounts for greater than 90% of the variation in plasma lipoprotein(a) concentrations. J Clin Invest.

[18] Marcovina SM, Viney NJ, Hughes SG, Xia S, Witztum JL (2018). Temporal variability in lipoprotein(a) levels in patients enrolled in the placebo arms of IONIS-APO(a)Rx and IONIS-APO(a)-LRx antisense oligonucleotide clinical trials. J Clin Lipidol.

[19] Kraft HG, Lingenhel A, Pang RW, Delport R, Trommsdorff M (1996). Frequency distributions of apolipoprotein(a) kringle IV repeat alleles and their effects on lipoprotein(a) levels in Caucasian, Asian, and African populations: the distribution of null alleles is non-random. Eur J Hum Genet.

[20] Mehta A, Jain V, Saeed A, Saseen JJ, Gulati M (2022). Lipoprotein(a) and ethnicities. Atherosclerosis.

[21] Sandholzer C, Hallman DM, Saha N, Sigurdsson G, Lackner C (1991). Effects of the apolipoprotein(a) size polymorphism on the lipoprotein(a) concentration in 7 ethnic groups. Hum Genet.

[22] Lee S, Prasad A, Choi Y, Xing C, Clopton P (2017). LPA gene, ethnicity, and cardiovascular events. Circulation.

[23] Deo RC, Wilson JG, Xing C, Lawson K, Kao WHL (2011). Single-nucleotide polymorphisms in LPA explain most of the ancestry-specific variation in Lp(a) levels in African Americans. PLoS One.

[24] Mukamel RE, Handsaker RE, Sherman MA, Barton AR, Zheng Y (2021). Protein-coding repeat polymorphisms strongly shape diverse human phenotypes. Science.

[25] Surakka I, Horikoshi M, Magi R, Sarin A-P, Mahajan A (2015). The impact of low-frequency and rare variants on lipid levels. Nat Genet.

[26] Graham SE, Clarke SL, Wu K-HH, Kanoni S, Zajac GJM (2021). The power of genetic diversity in genome-wide association studies of lipids. Nature.

[27] White AL, Hixson JE, Rainwater DL, Lanford RE (1994). Molecular basis for “null” lipoprotein(a) phenotypes and the influence of apolipoprotein(a) size on plasma lipoprotein(a) level in the baboon. J Biol Chem.

[28] Brunner C, Lobentanz EM, Pethö-Schramm A, Ernst A, Kang C (1996). The number of identical kringle IV repeats in apolipoprotein(a) affects its processing and secretion by HepG2 cells. J Biol Chem.

[29] Coassin S, Erhart G, Weissensteiner H, Araújo MEGde, Lamina C (2017). A novel but frequent variant in LPA KIV-2 is associated with a pronounced Lp(a) and cardiovascular risk reduction. Eur Heart J.

[30] Schachtl-Riess JF, Kheirkhah A, Grüneis R, Di Maio S, Schoenherr S (2021). Frequent LPA KIV-2 variants lower lipoprotein(a) concentrations and protect against coronary artery disease. J Am Coll Cardiol.

[31] Kronenberg F, Kuen E, Ritz E, Junker R, Konig P (2000). Lipoprotein(a) serum concentrations and apolipoprotein(a) phenotypes in mild and moderate renal failure. J Am Soc Nephrol.

[32] Enkhmaa B, Anuurad E, Zhang W, Kim K, Berglund L (2019). Heritability of apolipoprotein (a) traits in two-generational African-American and Caucasian families. J Lipid Res.

[33] Sandholzer C, Saha N, Kark JD, Rees A, Jaross W (1992). Apo(a) isoforms predict risk for coronary heart disease. A study in six populations. Arterioscler Thromb.

[34] Kraft HG, Lingenhel A, Kochl S, Hoppichler F, Kronenberg F (1996). Apolipoprotein(a) kringle IV repeat number predicts risk for coronary heart disease. Arterioscler Thromb Vasc Biol.

[35] Lamina C (2022). Mendelian randomization - principles and its usage in lipoprotein(a) research. Atherosclerosis.

[36] Ridker PM, Hennekens CH, Stampfer MJ (1993). A prospective study of lipoprotein (a) and the risk of myocardial infarction. JAMA.

[37] Erqou S, Thompson A, Di Angelantonio E, Saleheen D, Kaptoge S (2010). Apolipoprotein(a) isoforms and the risk of vascular disease: systematic review of 40 studies involving 58,000 participants. J Am Coll Cardiol.

[38] Gudbjartsson DF, Thorgeirsson G, Sulem P, Helgadottir A, Gylfason A (2019). Lipoprotein(a) concentration and risks of cardiovascular disease and diabetes. J Am Coll Cardiol.

[39] Clarke R, Peden JF, Hopewell JC, Kyriakou T, Goel A (2009). Genetic variants associated with Lp(a) lipoprotein level and coronary disease. N Engl J Med.

[40] Kamstrup PR, Tybjaerg-Hansen A, Steffensen R, Nordestgaard BG (2009). Genetically elevated lipoprotein(a) and increased risk of myocardial infarction. JAMA.

[41] Kamstrup PR, Tybjærg-Hansen A, Nordestgaard BG (2013). Extreme lipoprotein(a) levels and improved cardiovascular risk prediction. J Am Coll Cardiol.

[42] Patel AP, Wang M, Pirruccello JP, Ellinor PT, Ng K (2021). Lp(a) (Lipoprotein [a]) concentrations and incident atherosclerotic cardiovascular disease. Arterioscler Thromb Vasc Biol.

[43] Trinder M, Uddin MM, Finneran P, Aragam KG, Natarajan P (2020). Clinical utility of lipoprotein(a) and LPA genetic risk score in risk prediction of incident atherosclerotic cardiovascular disease. JAMA Cardiol.

[44] Wu H, Luan J, Forgetta V, Engert JC, Thanassoulis G (2021). Utility of genetically predicted Lp(a) (lipoprotein [a]) and ApoB levels for cardiovascular risk assessment. Circ Genomic Precis Med.

[45] Kyriakou T, Seedorf U, Goel A, Hopewell JC, Clarke R (2014). A common LPA null allele associates with lower lipoprotein(a) levels and coronary artery disease risk. Arterioscler Thromb Vasc Biol.

[46] Emdin CA, Khera AV, Natarajan P, Klarin D, Won H-H (2016). Phenotypic characterization of genetically lowered human lipoprotein(a) levels. J Am Coll Cardiol.

[47] Mora S, Kamstrup PR, Rifai N, Nordestgaard BG, Buring JE (2010). Lipoprotein(a) and risk of type 2 diabetes. Clin Chem.

[48] Kamstrup PR, Nordestgaard BG (2013). Lipoprotein(a) concentrations, isoform size, and risk of type 2 diabetes: a Mendelian randomisation study. Lancet Diabetes Endocrinol.

[49] Lamina C, Kronenberg F (2013). The mysterious lipoprotein(a) is still good for a surprise. Lancet Diabetes Endocrinol.

[50] Lamina C, Ward NC (2022). Lipoprotein(a) and diabetes mellitus. Atherosclerosis.

[51] Kraft HG, Windegger M, Menzel HJ, Utermann G (1998). Significant impact of the + 93 C/T polymorphism in the apolipoprotein(a) gene on Lp(a) concentrations in Africans but not in Caucasians: confounding effect of linkage disequilibrium. Hum Mol Genet.

[52] Enkhmaa B, Anuurad E, Berglund L (2016). Lipoprotein (a): impact by ethnicity and environmental and medical conditions. J Lipid Res.

[53] Noureen A, Ronke C, Khalifa M, Halbwax M, Fischer A (2017). Significant differentiation in the apolipoprotein(a)/lipoprotein(a) trait between chimpanzees from Western and Central Africa. Am J Primatol.

[54] Coassin S, Chemello K, Khantalin I, Forer L, Dottelmayer P Genome-Wide Characterization of a Highly Penetrant Form of Hyperlipoproteinemia Associated With Genetically Elevated Cardiovascular Risk. Circ Genomic Precis Med in press.

[55] Langsted A, Nordestgaard BG, Kamstrup PR (2021). Low lipoprotein(a) levels and risk of disease in a large, contemporary, general population study. Eur Heart J.

[56] King A (2018). A CRISPR edit for heart disease. Nature.

[57] Coassin S, Schönherr S, Weissensteiner H, Erhart G, Forer L (2019). A comprehensive map of single-base polymorphisms in the hypervariable LPA kringle IV type 2 copy number variation region. J Lipid Res.

[58] Byrne CD, Schwartz K, Meer K, Cheng JF, Lawn RM (1994). The human apolipoprotein(a)/plasminogen gene cluster contains a novel homologue transcribed in liver. Arterioscler Thromb.

[59] Beck CR, Garcia-Perez JL, Badge RM, Moran JV (2011). LINE-1 elements in structural variation and disease. Annu Rev Genom Hum Genet.

[60] Bourque G, Burns KH, Gehring M, Gorbunova V, Seluanov A (2018). Ten things you should know about transposable elements. Genome Biol.

[61] Lackner C, Cohen JC, Hobbs HH (1993). Molecular definition of the extreme size polymorphism in apolipoprotein(a). Hum Mol Genet.

[62] Rosby O, Aleströom P, Berg K (2000). Sequence conservation in kringle IV-type 2 repeats of the LPA gene. Atherosclerosis.

[63] Parson W, Kraft HG, Niederstaötter H, Lingenhel AW, Koöchl S (2004). A common nonsense mutation in the repetitive Kringle IV-2 domain of human apolipoprotein(a) results in a truncated protein and low plasma Lp(a). Hum Mutat.

[64] Noureen A, Fresser F, Utermann G, Schmidt K (2015). Sequence variation within the KIV-2 copy number polymorphism of the human LPA gene in african, asian, and European populations. PLoS One.

[65] Waldeyer C, Makarova N, Zeller T, Schnabel RB, Brunner FJ (2017). Lipoprotein(a) and the risk of cardiovascular disease in the European population: results from the BiomarCaRE consortium. Eur Heart J.

[66] Wade DP, Lindahl GE, Lawn RM (1994). Apolipoprotein(a) gene transcription is regulated by liver-enriched trans-acting factor hepatocyte nuclear factor 1 alpha. J Biol Chem.

[67] Acquati F, Hammer R, Ercoli B, Mooser V, Tao R (1999). Transgenic mice expressing a human apolipoprotein[a] allele. J Lipid Res.

[68] Bopp S, Köchl S, Acquati F, Magnaghi P, Pethö-Schramm A (1995). Ten allelic apolipoprotein[a] 5’ flanking fragments exhibit comparable promoter activities in HepG2 cells. J Lipid Res.

[69] Hoover-Plow J, Huang M (2013). Lipoprotein(a) metabolism: potential sites for therapeutic targets. Metabolism.

[70] Negi S, Singh SK, Pati N, Handa V, Chauhan R (2004). A proximal tissue-specific module and a distal negative regulatory module control apolipoprotein (a) gene transcription. Biochem J.

[71] Chennamsetty I, Claudel T, Kostner KM, Trauner M, Kostner GM (2012). FGF19 signaling cascade suppresses APOA gene expression. Arterioscler Thromb Vasc Biol.

[72] Magnaghi P, Mihalich A, Taramelli R (1994). Several liver-specific DNase hypersensitive sites are present in the intergenic region separating human plasminogen and apoprotein(A) genes. Biochem Biophys Res Commun.

[73] Wade DP, Puckey LH, Knight BL, Acquati F, Mihalich A (1997). Characterization of multiple enhancer regions upstream of the apolipoprotein(a) gene. J Biol Chem.

[74] Puckey LH, Knight BL (2002). Interaction of oestrogen and peroxisome proliferator-activated receptors with apolipoprotein(a) gene enhancers. Biochem J.

[75] Zídková K, Kebrdlová V, Zlatohlávek L, Ceska R (2007). Detection of variability in apo (a) gene transcription regulatory sequences using the DGGE method. Clin Chim Acta.

[76] Puckey LH, Knight BL (2003). Sequence and functional changes in a putative enhancer region upstream of the apolipoprotein(a) gene. Atherosclerosis.

[77] Mack S, Coassin S, Rueedi R, Yousri NA, Seppöalaö I (2017). A genome-wide association meta-analysis on lipoprotein (a) concentrations adjusted for apolipoprotein (a) isoforms. J Lipid Res.

[78] Buniello A, MacArthur JAL, Cerezo M, Harris LW, Hayhurst J (2019). The NHGRI-EBI GWAS Catalog of published genome-wide association studies, targeted arrays and summary statistics 2019. Nucleic Acids Res.

[79] Perombelon YFN, Soutar AK, Knight BL (1994). Variation in lipoprotein(a) concentration associated with different apolipoprotein(a) alleles. J Clin Invest.

[80] Rainwater DL (1995). Genetic basis for multimodal relationship between apolipoprotein (a) size and lipoprotein (a) concentration in Mexican-Americans. Atherosclerosis.

[81] Rainwater DL, Kammerer CM, VandeBerg JL, Hixson JE (1997). Characterization of the genetic elements controlling lipoprotein(a) concentrations in Mexican Americans. Evidence for at least three controlling elements linked to LPA, the locus encoding apolipoprotein(a). Atherosclerosis.

[82] Cohen JC, Chiesa G, Hobbs HH (1993). Sequence polymorphisms in the apolipoprotein (a) gene. Evidence for dissociation between apolipoprotein(a) size and plasma lipoprotein(a) levels. J Clin Invest.

[83] Mooser V, Mancini FP, Bopp S, Pethoö-Schramm A, Guerra R (1995). Sequence polymorphisms in the apo(a) gene associated with specific levels of Lp(a) in plasma. Hum Mol Genet.

[84] Rubin J, Kim HJ, Pearson TA, Holleran S, Ramakrishnan R (2006). Apo[a] size and PNR explain African American-Caucasian differences in allele-specific apo[a] levels for small but not large apo[a]. J Lipid Res.

[85] Erhart G, Lamina C, Lehtimaki T, Marques-Vidal P, Köhönen M (2018). Genetic factors explain a major fraction of the 50% lower lipoprotein(a) concentrations in Finns. Arterioscler Thromb Vasc Biol.

[86] Mancini FP, Mooser V, Guerra R, Hobbs HH (1995). Sequence microheterogeneity in apolipoprotein(a) gene repeats and the relationship to plasma Lp(a) levels. Hum Mol Genet.

[87] Prins J, Leus FR, Bouma BN, van Rijn HJ (1999). The identification of polymorphisms in the coding region of the apolipoprotein (a) gene-association with earlier identified polymorphic sites and influence on the lipoprotein (a) concentration. Thromb Haemostasis.

[88] Puckey L (1997). Polymorphisms in the apolipoprotein(a) gene and their relationship to allele size and plasma lipoprotein(a) concentration. Hum Mol Genet.

[89] Mooser V, Seabra MC, Abedin M, Landschulz KT, Marcovina S (1996). Apolipoprotein(a) kringle 4-containing fragments in human urine. Relationship to plasma levels of lipoprotein(a). J Clin Invest.

[90] Luke MM, Kane JP, Liu DM, Rowland CM, Shiffman D (2007). A polymorphism in the protease-like domain of apolipoprotein(a) is associated with severe coronary artery disease. Arterioscler Thromb Vasc Biol.

[91] Kraft HG, Haibach C, Lingenhel A, Brunner C, Trommsdorff M (1995). Sequence polymorphism in kringle IV 37 in linkage disequilibrium with the apolipoprotein (a) size polymorphism. Hum Genet.

[92] Hirschfeldova K, Lipovska D, Skrha P, Ceska R (2009). The apo(a) gene (TTTTA)n promoter polymorphism and its association with variability in exons of the kringle IV types 8 to 10. Clin Chim Acta.

[93] Lanktree MB, Anand SS, Yusuf S, Hegele RA (2010). Share Investigators, Comprehensive analysis of genomic variation in the LPA locus and its relationship to plasma lipoprotein(a) in South Asians, Chinese, and European Caucasians. Circ Cardiovasc Genet.

[94] Ogorelkova M, Kraft HG, Ehnholm C, Utermann G (2001). Single nucleotide polymorphisms in exons of the apo(a) kringles IV types 6 to 10 domain affect Lp (a) plasma concentrations and have different patterns in Africans and Caucasians. Hum Mol Genet.

[95] Crawford DC, Peng Z, Cheng J-F, Boffelli D, Ahearn M (2008). LPA and PLG sequence variation and kringle IV-2 copy number in two populations. Hum Hered.

[96] Di Maio S, Grüneis R, Streiter G, Lamina C, Maglione M (2020). Investigation of a nonsense mutation located in the complex KIV-2 copy number variation region of apolipoprotein(a) in 10,910 individuals. Genome Med.

[97] Scanu AM, Pfaffinger D, Lee JC, Hinman J (1994). A single point mutation (Trp72->Arg) in human apo(a) kringle 4-37 associated with a lysine binding defect in Lp (a). Biochim Biophys Acta.

[98] Scanu AM, Miles LA, Fless GM, Pfaffinger D, Eisenbart J (1993). Rhesus monkey lipoprotein(a) binds to lysine Sepharose and U937 monocytoid cells less efficiently than human lipoprotein(a). Evidence for the dominant role of kringle 4 (37). J Clin Invest.

[99] Edelstein C, Mandala M, Pfaffinger D, Scanu AM (1995). Determinants of lipoprotein (a) assembly: a study of wild-type and mutant apolipoprotein(a) phenotypes isolated from human and rhesus monkey lipoprotein(a) under mild reductive conditions. Biochemistry.

[100] Chretien J-P, Coresh J, Berthier-Schaad Y, Kao WHL, Fink NE (2006). Three single-nucleotide polymorphisms in LPA account for most of the increase in lipoprotein(a) level elevation in African Americans compared with European Americans. J Med Genet.

[101] Zysow BR, Lindahl GE, Wade DP, Knight BL, Lawn RM (1995). C/T polymorphism in the 5’ untranslated region of the apolipoprotein(a) gene introduces an upstream ATG and reduces in vitro translation. Arterioscler Thromb Vasc Biol.

[102] Suzuki K, Kuriyama M, Saito T, Ichinose A (1997). Plasma lipoprotein(a) levels and expression of the apolipoprotein(a) gene are dependent on the nucleotide polymorphisms in its 5’-flanking region. J Clin Invest.

[103] Ichinose A, Kuriyama M (1995). Detection of polymorphisms in the 5’-flanking region of the gene for apolipoprotein(a). Biochem Biophys Res Commun.

[104] Zeng L, Moser S, Mirza-Schreiber N, Lamina C, Coassin S (2022). Cis-epistasis at the LPA locus and risk of cardiovascular diseases. Cardiovasc Res.

[105] Kronenberg F (2014). Genetic determination of lipoprotein(a) and its association with cardiovascular disease: convenient does not always mean better. J Intern Med.

[106] Coassin S, Hermann-Kleiter N, Haun M, Wahl S, Wilson R (2020). A genome-wide analysis of DNA methylation identifies a novel association signal for Lp(a) concentrations in the LPA promoter. PLoS One.

[107] Jones GT, Marsman J, Bhat B, Phillips VL, Chatterjee A (2020). DNA methylation profiling identifies a high effect genetic variant for lipoprotein(a) levels. Epigenetics.

[108] Said MA, Yeung MW, van de Vegte YJ, Benjamins JW, Dullaart RPF (2021). Genome-wide association study and identification of a protective missense variant on lipoprotein(a) concentration. Arterioscler Thromb Vasc Biol.

[109] Kronenberg F (2019). Prediction of cardiovascular risk by Lp(a) concentrations or genetic variants within the LPA gene region. Clin Res Cardiol Suppl.

[110] Lobentanz EM, Krasznai K, Gruber A, Brunner C, Muller HJ (1998). Intracellular metabolism of human apolipoprotein(a) in stably transfected Hep G2 cells. Biochemistry.

[111] Cox LA, Jett C, Hixson JE (1998). Molecular basis of an apolipoprotein[a] null allele: a splice site mutation is associated with deletion of a single exon. J Lipid Res.

[112] Morgan BM, Brown AN, Deo N, Harrop TWR, Taiaroa G (2020). Nonsynonymous SNPs in LPA homologous to plasminogen deficiency mutants represent novel null apo(a) alleles. J Lipid Res.

[113] Ogorelkova M, Gruber A, Utermann G (1999). Molecular basis of congenital Lp(a) deficiency: a frequent apo(a) “null” mutation in caucasians. Hum Mol Genet.

[114] Tybjærg-Hansen A (2016). Using human genetics to predict the effects and side-effects of drugs. Curr Opin Lipidol.

[115] Lim ET, Würtz P, Havulinna AS, Palta P, Tukiainen T (2014). Distribution and medical impact of loss-of-function variants in the Finnish founder population. PLoS Genet.

[116] Karczewski KJ, Francioli LC, Tiao G, Cummings BB, Alfoöldi J (2020). The mutational constraint spectrum quantified from variation in 141,456 humans. Nature.

[117] Taliun D, Harris DN, Kessler MD, Carlson J, Szpiech ZA (2021). Sequencing of 53,831 diverse genomes from the NHLBI TOPMed Program. Nature.

[118] Rosby O, Alestroöm P, Berg K (1997). High-degree sequence conservation in LPA kringle IV-type 2 exons and introns. Clin Genet.

[119] Harismendy O, Schwab RB, Bao L, Olson J, Rozenzhak S (2011). Detection of low prevalence somatic mutations in solid tumors with ultra-deep targeted sequencing. Genome Biol.

[120] Kronenberg F (2022). Lipoprotein(a) measurement issues: are we making a mountain out of a Molehill?. Atherosclerosis.

[121] Contois JH, Wu AHB, Li Z, Feroze AH, Grunenberger F (2000). Distribution of serum apolipoproteins A-I and B and lipoprotein(a) in European elderly. The SENECA study. Clin Chim Acta.

[122] Li Y, Luke MM, Shiffman D, Devlin JJ (2011). Genetic variants in the apolipoprotein (a) gene and coronary heart disease. Circ Cardiovasc Genet.

[123] Khalifa M, Noureen A, Ertelthalner K, Bandegi AR, Delport R (2015). Lack of association of rs3798220 with small apolipoprotein(a) isoforms and high lipoprotein(a) levels in East and Southeast Asians. Atherosclerosis.

[124] Utermann G (1999). Genetic architecture and evolution of the lipoprotein(a) trait. Curr Opin Lipidol.

[125] Melzer D, Perry JRB, Hernandez D, Corsi A-M, Stevens K (2008). A genome-wide association study identifies protein quantitative trait loci (pQTLs). PLoS Genet.

[126] Ober C, Nord AS, Thompson EE, Pan L, Tan Z (2009). Genome-wide association study of plasma lipoprotein(a) levels identifies multiple genes on chromosome 6q. J Lipid Res.

[127] Trégouet D-A, Konig IR, Erdmann J, Munteanu A, Braund PS (2009). Genome-wide haplotype association study identifies the SLC22A3-LPAL2-LPA gene cluster as a risk locus for coronary artery disease. Nat Genet.

[128] Lu W, Cheng Y-C, Chen K, Wang H, Gerhard GS (2015). Evidence for several independent genetic variants affecting lipoprotein (a) cholesterol levels. Hum Mol Genet.

[129] Li J, Lange LA, Sabourin J, Duan Q, Valdar W (2015). Genome- and exome-wide association study of serum lipoprotein (a) in the Jackson Heart Study. J Hum Genet.

[130] Zekavat SM, Ruotsalainen S, Handsaker RE, Alver M, Bloom J (2018). Deep coverage whole genome sequences and plasma lipoprotein(a) in individuals of European and African ancestries. Nat Commun.

[131] Hoekstra M, Chen HY, Rong J, Dufresne L, Yao J (2021). Genome-wide association study highlights APOH as a novel locus for lipoprotein(a) levels. Arterioscler Thromb Vasc Biol.

[132] Zabaneh D, Kumari M, Sandhu M, Wareham N, Wainwright N (2011). Meta analysis of candidate gene variants outside the LPA locus with Lp(a) plasma levels in 14,500 participants of six White European cohorts. Atherosclerosis.

[133] Burgess S, Ference BA, Staley JR, Freitag DF, Mason AM (2018). Association of LPA variants with risk of coronary disease and the implications for lipoprotein (a)-lowering therapies: a mendelian randomization analysis. JAMA Cardiol.

[134] Dron JS, Wang M, Patel AP, Kartoun U, Ng K (2021). Genetic predictor to identify individuals with high lipoprotein(a) concentrations. Circ Genomic Precis Med.

[135] Bycroft C, Freeman C, Petkova D, Band G, Elliott LT (2018). The UK Biobank resource with deep phenotyping and genomic data. Nature.

[136] Mak ACY, Lai YYY, Lam ET, Kwok TP, Leung AKY (2016). Genome-wide structural variation detection by genome mapping on nanochannel arrays. Genetics.

[137] Erdel M, Hubalek M, Lingenhel A, Kofler K, Duba HC (1999). Counting the repetitive kringle-IV repeats in the gene encoding human apolipoprotein(a) by fibre-FISH. Nat Genet.

[138] McCormick SPA, Schneider WJ (2019). Lipoprotein(a) catabolism: a case of multiple receptors. Pathology.

[139] Chemello K, Chan DC, Lambert G, Watts GF (2022). Recent advances in demystifying the metabolism of lipoprotein(a). Atherosclerosis.

[140] Yang X, Sethi A, Yanek LR, Knapper C, Nordestgaard BG (2016). SCARB1 gene variants are associated with the phenotype of combined high high-density lipoprotein cholesterol and high lipoprotein (a). Circ Cardiovasc Genet.

[141] Visscher PM, Wray NR, Zhang Q, Sklar P, McCarthy MI (2017). 10 Years of GWAS discovery: biology, function, and translation. Am J Hum Genet.

[142] Köchl S, Fresser F, Lobentanz E, Baier G, Utermann G (1997). Novel interaction of apolipoprotein(a) with beta-2 glycoprotein I mediated by the kringle IV domain. Blood.

[143] van Capelleveen JC, van der Valk FM, Stroes ESG (2016). Current therapies for lowering lipoprotein (a). J Lipid Res.

[144] Lambert G, Thedrez A, Croyal M, Ramin-Mangata S, Couret D (2017). The complexity of lipoprotein (a) lowering by PCSK9 monoclonal antibodies. Clin Sci (Lond).

[145] Koschinsky ML, Boffa MB (2021). Genetics to the rescue: sophisticated approaches provide critical insights into the determination of Lp(a) levels. J Am Coll Cardiol.

[146] Wichmann H-E, Gieger C, Illig T, MONICA/KORA Study Group (2005). KORA-gen–resource for population genetics, controls and a broad spectrum of disease phenotypes. Gesundheitswesen.

[147] Sudmant PH, Rausch T, Gardner EJ, Handsaker RE, Abyzov A (2015). An integrated map of structural variation in 2,504 human genomes. Nature.

[148] Stefanutti C, Pisciotta L, Favari E, Bopp S, Vacondio F (2020). Lipoprotein (a) concentration, genetic variants, apo(a) isoform size, and cellular cholesterol efflux in patients with elevated Lp(a) and coronary heart disease submitted or not to lipoprotein apheresis: an Italian case-control multicenter study on Lp(a). J Clin Lipidol.

[149] Zídkova K, Zlatohlávek L, Ceska R (2007). Variability in apo(a) gene regulatory sequences, compound genotypes, and association with Lp(a) plasma levels. Clin Biochem.

[150] Qi Q, Workalemahu T, Zhang C, Hu FB, Qi L (2012). Genetic variants, plasma lipoprotein(a) levels, and risk of cardiovascular morbidity and mortality among two prospective cohorts of type 2 diabetes. Eur Heart J.

[151] Postmus I, Trompet S, Deshmukh HA, Barnes MR, Li X (2014). Pharmacogenetic meta-analysis of genome-wide association studies of LDL cholesterol response to statins. Nat Commun.

[152] McInnes G, Tanigawa Y, DeBoever C, Lavertu A, Olivieri JE (2020). Global Biobank engine. Phenotype: lipoprotein A covariate and statin adjusted.

[153] Wu JH, Lee IN (2003). Studies of apolipoprotein (a) promoter from subjects with different plasma lipoprotein (a) concentrations. Clin Biochem.

[154] Wade DP, Clarke JG, Lindahl GE, Liu AC, Zysow BR (1993). 5’ control regions of the apolipoprotein(a) gene and members of the related plasminogen gene family. Proc Natl Acad Sci Unit States Am.

[155] Trommsdorff M, Köchl S, Lingenhel A, Kronenberg F, Delport R (1995). A pentanucleotide repeat polymorphism in the 5’ control region of the apolipoprotein(a) gene is associated with lipoprotein(a) plasma concentrations in Caucasians. J Clin Invest.

[156] van der Hoek YY, Wittekoek ME, Beisiegel U, Kastelein JJP, Koschinsky ML (1993). The apolipoprotein(a) kringle IV repeats which differ from the major repeat kringle are present in variably-sized isoforms. Hum Mol Genet.

[157] Prins J, van der Hoek YY, Biesheuvel TH, Leus FR, van Rijn HJM (1998). The functional and clinical significance of the Met^Thr substitution in kringle IV type 10 of apolipoprotein(a). Thromb Res.

[158] Pfaffinger D, Lean JM, Scanu AM (1993). Amplification of human APO(a) kringel 4-37 from blood lymphocyte DNA. Biochim Biophys Acta (BBA) - Mol Basis Dis.

[159] Arai K, Luke MM, Koschinsky ML, Miller ER, Pullinger CR (2010). The I4399M variant of apolipoprotein(a) is associated with increased oxidized phospholipids on apolipoprotein B-100 particles. Atherosclerosis.

[160] Scipione CA, McAiney JT, Simard DJ, Bazzi ZA, Gemin M (2017). Characterization of the I4399M variant of apolipoprotein(a): implications for altered prothrombotic properties of lipoprotein(a). J Thromb Haemostasis.

[161] Rowland CM, Pullinger CR, Luke MM, Shiffman D, Green L (2014). Lipoprotein (a), LPA Ile4399Met, and fibrin clot properties. Thromb Res.

