# Supplementary material for: Lipoprotein(a) beyond the kringle IV repeat polymorphism: The complexity of genetic variation in the LPA gene
Source: Atherosclerosis. Author manuscript; Available in PMC 2022 Sep 15. (PMC7613587; doi:10.1016/j.atherosclerosis.2022.04.003)
Supplement: Supplementary File [file EMS152740-supplement-Supplementary_File.pdf]

Supplemental Materials to

Lipoprotein(a) beyond the kringle IV repeat polymorphism: the complexity of genetic variation in the *LPA* gene

Stefan Coassin, Florian Kronenberg

Supplemental Figures

Supplemental Figure 1. Per-base identity between the exons of *LPA* and between the intronic context sequences.

The values indicate the percentage of identical bases in pairwise alignments of the eleven kringle IV domains (including KIV-2B) and the kringle V. The color code ranges from green (low identity) to dark red (high identity). All KIV share extensive homologies, which tend to decrease with increasing distance from each other, remaining, however, still >70% and often >85%. The intronic alignments are based on ±200 bp intronic context sequences in each direction of the respective exon.

Figure taken from Coassin S, Schönherr S, et al, J Lipid Res 60:186-199, 2019[1] (Supplemental Figure 12A).

Coding sequence – 1st kringle exon

	K 4-1	K 4-2	K 4-2B	K 4-3	K 4-4	K 4-5	K 4-6	K 4-7	K 4-8	K 4-9	K 4-10	K 5
K 4-1	100	88	86	86	84	84	84	83	79	89	86	53
K 4-2	88	100	98	98	95	89	84	83	83	87	86	51
K 4-2B	86	98	100	100	94	89	82	81	82	85	84	51
K 4-3	86	98	100	100	94	89	82	81	82	85	84	51
K 4-4	84	95	94	94	100	86	80	79	80	83	83	46
K 4-5	84	89	89	89	86	100	79	79	78	82	83	50
K 4-6	84	84	82	82	80	79	100	94	85	88	87	49
K 4-7	83	83	81	81	79	79	94	100	84	87	87	51
K 4-8	79	83	82	82	80	78	85	84	100	84	83	50
K 4-9	89	87	85	85	83	82	88	87	84	100	90	48
K 4-10	86	86	84	84	83	83	87	87	83	90	100	51
K 5	53	51	51	51	46	50	49	51	50	48	51	100

Coding sequence – 2nd kringle exon

	K 4-1	K 4-2	K 4-2B	K 4-3	K 4-4	K 4-5	K 4-6	K 4-7	K 4-8	K 4-9	K 4-10	K 5
K 4-1	100	100	100	96	85	75	72	76	79	75	84	54
K 4-2	100	100	100	96	85	75	72	76	79	75	84	54
K 4-2B	100	100	100	96	85	75	72	76	79	75	84	54
K 4-3	96	96	96	100	86	76	71	75	80	75	83	53
K 4-4	85	85	85	86	100	74	70	72	76	75	79	56
K 4-5	75	75	75	76	74	100	87	91	92	81	77	63
K 4-6	72	72	72	71	70	87	100	86	85	77	72	63
K 4-7	76	76	76	75	72	91	86	100	92	77	79	63
K 4-8	79	79	79	80	76	92	85	92	100	80	81	64
K 4-9	75	75	75	75	75	81	77	77	80	100	72	57
K 4-10	84	84	84	83	79	77	72	79	81	72	100	58
K 5	54	54	54	53	56	63	63	63	64	57	58	100

Intronic context sequence (±200 bp) – of 1st kringle exon

	K4-1	K4-2	K4-2B	K4-3	K4-4	K4-5	K4-6	K4-7	K4-8	K4-9	K4-10	K5
K 4-1	100	69	68	68	70	59	69	71	70	69	71	39
K 4-2	69	100	94	90	77	68	59	62	60	62	64	38
K 4-2B	68	94	100	96	77	66	58	62	61	62	63	38
K 4-3	68	90	96	100	78	67	60	63	63	64	64	37
K 4-4	70	77	77	78	100	65	59	63	64	65	62	39
K 4-5	59	68	66	67	65	100	54	55	55	52	54	37
K 4-6	69	59	58	60	59	54	100	86	65	65	66	34
K 4-7	71	62	62	63	63	55	86	100	68	69	68	35
K 4-8	70	60	61	63	64	55	65	68	100	72	69	37
K 4-9	69	62	62	64	65	52	65	69	72	100	76	35
K 4-10	71	64	63	64	62	54	66	68	69	76	100	36
K 5	39	38	38	37	39	37	34	35	37	35	36	100

Intronic context sequence (±200 bp) – of 2nd kringle exon

	K4-1	K4-2	K4-2B	K4-3	K4-4	K4-5	K4-6	K4-7	K4-8	K4-9	K4-10	K5
K 4-1	100	100	100	94	81	64	67	61	72	71	71	39
K 4-2	100	100	100	94	81	64	67	61	72	71	71	39
K 4-2B	100	100	100	94	81	64	67	61	72	71	71	39
K 4-3	94	94	94	100	80	65	68	62	73	71	72	39
K 4-4	81	81	81	80	100	63	67	61	73	69	71	40
K 4-5	64	64	64	65	63	100	88	83	79	74	70	39
K 4-6	67	67	67	68	67	88	100	84	78	76	72	41
K 4-7	61	61	61	62	61	83	84	100	73	70	68	40
K 4-8	72	72	72	73	73	79	78	73	100	78	77	41
K 4-9	71	71	71	71	69	74	76	70	78	100	74	43
K 4-10	71	71	71	72	71	70	72	68	77	74	100	44
K 5	39	39	39	39	40	39	41	40	41	43	44	100

Supplemental Figure 2. SNP haplotypes defining the KIV-2 types A, B and C.

The KIV-2 types A, B and C are defined by three synonymous SNPs at positions 14, 41 and 86 of the first KIV-2 exon[1–4]. However, >100 differences exist in the introns. A T/C SNP 119 bp downstream of exon 1 (position 859 in [1]) has been recently used to split the KIV-2A subtypes in two further subtypes[5].

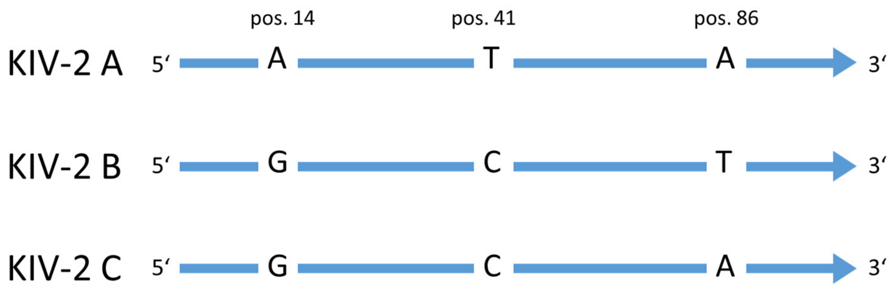

Supplemental Figure 3. Principle of KIV-2 batch sequencing

LPA KIV-2 batch sequencing uses primer that bind in all KIV-2 repeats to amplify all units in one amplicon mixture. This mixture is then sequenced using ultra-deep next generation sequencing (>1000x). The repetitive structure of the KIV-2 repeat prevents a unique alignment of sequencing reads. Common alignment algorithms locate such reads to the region, but assign a low mapping quality[6]. The reads are then disregarded by variant calling tools (right lower panel)[6]. This can be alleviated by aligning all reads to a single repeat[1,4,7]. Variant bases from the same position originating from one or only a subset of kringles (orange circles) will be represented only in a respective fraction of reads, with the wild type base (blue circle) representing the major fraction. Such variants are not called by common germline variants callers as they are employed by large genome reference datasets (1000 Genomes[8], GnomAD[9], TOPMed[10]), but they can be retrieved using variant callers optimized for calling of somatic mutations or mitochondrial heteroplasmies[1,11]. This procedure allows identifying variants from the KIV-2 region with very high sensitivity[1]. However, it does not allow allocating a variant to a specific repeat within the region.

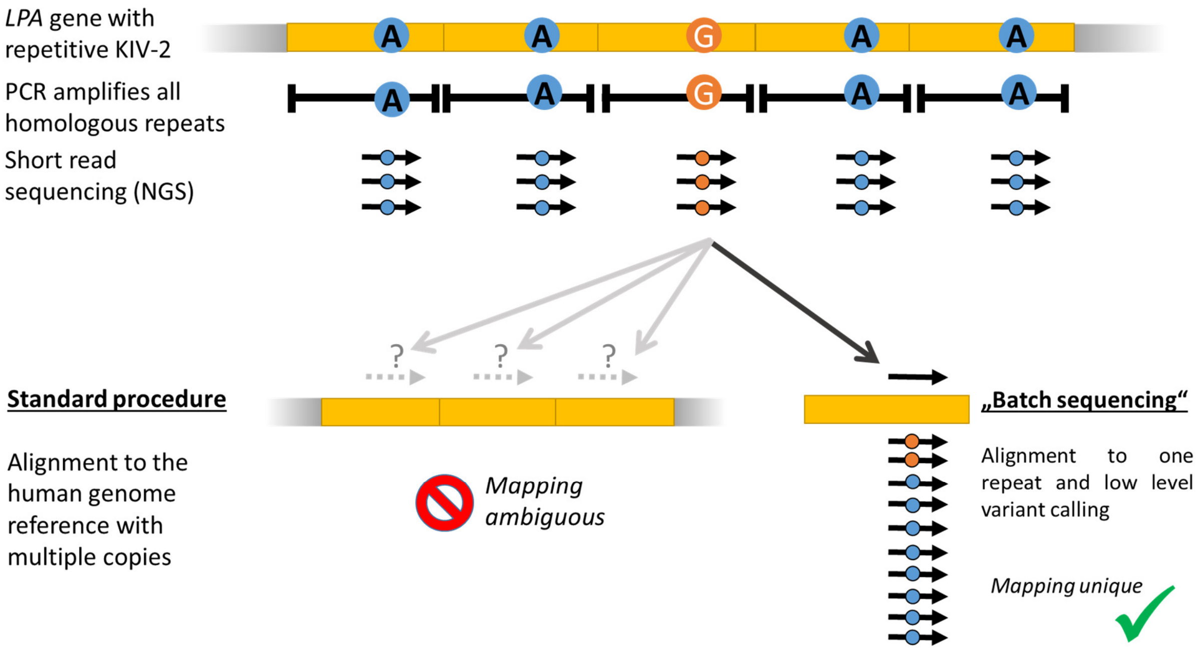

References

[1] S. Coassin, S. Schönherr, H. Weissensteiner, G. Erhart, L. Forer, et al., A comprehensive map of single-base polymorphisms in the hypervariable LPA kringle IV type 2 copy number variation region., J Lipid Res. 60 (2019) 186–199. https://doi.org/10.1194/jlr.M090381.

[2] J.W. McLean, J.E. Tomlinson, W.J. Kuang, D.L. Eaton, E.Y. Chen, et al., cDNA sequence of human apolipoprotein(a) is homologous to plasminogen., Nature. 330 (1987) 132–7. https://doi.org/10.1038/330132a0.

[3] W. Parson, H.G. Kraft, H. Niederstätter, A.W. Lingenhel, S. Köchl, et al., A common nonsense mutation in the repetitive Kringle IV-2 domain of human apolipoprotein(a) results in a truncated protein and low plasma Lp(a)., Hum Mutat. 24 (2004) 474–80. https://doi.org/10.1002/humu.20101.

[4] A. Noureen, F. Fresser, G. Utermann, K. Schmidt, Sequence Variation within the KIV-2 Copy Number Polymorphism of the Human LPA Gene in African, Asian, and European Populations, PLoS One. 10 (2015) e0121582. https://doi.org/10.1371/journal.pone.0121582.

[5] R.E. Mukamel, R.E. Handsaker, M.A. Sherman, A.R. Barton, Y. Zheng, et al., Protein-coding repeat polymorphisms strongly shape diverse human phenotypes, Science. 373 (2021) 1499–1505. https://doi.org/10.1126/science.abg8289.

[6] M.T.W. Ebbert, T.D. Jensen, K. Jansen-West, J.P. Sens, J.S. Reddy, et al., Systematic analysis of dark and camouflaged genes reveals disease-relevant genes hiding in plain sight., Genome Biol. 20 (2019) 97. https://doi.org/10.1186/s13059-019-1707-2.

[7] O. Rosby, P. Aleström, K. Berg, High-degree sequence conservation in LPA kringle IV-type 2 exons and introns, Clin Genet. 52 (1997) 293–302. http://onlinelibrary.wiley.com/doi/10.1111/j.1399-0004.1997.tb04346.x/pdf.

[8] P.H. Sudmant, T. Rausch, E.J. Gardner, R.E. Handsaker, A. Abyzov, et al., An integrated map of structural variation in 2,504 human genomes, Nature. 526 (2015) 75–81. https://doi.org/10.1038/nature15394.

[9] K.J. Karczewski, L.C. Francioli, G. Tiao, B.B. Cummings, J. Alföldi, et al., The mutational constraint spectrum quantified from variation in 141,456 humans, Nature. 581 (2020) 434–443. https://doi.org/10.1038/s41586-020-2308-7.

[10] D. Taliun, D.N. Harris, M.D. Kessler, J. Carlson, Z.A. Szpiech, et al., Sequencing of 53,831 diverse genomes from the NHLBI TOPMed Program, Nature. 590 (2021) 290–299. https://doi.org/10.1038/s41586-021-03205-y.

[11] H. Weissensteiner, L. Forer, C. Fuchsberger, B. Schöpf, A. Kloss-Brandstätter, et al., mtDNA-Server: next-generation sequencing data analysis of human mitochondrial DNA in the cloud., Nucleic Acids Res. 44 (2016) W64-9. https://doi.org/10.1093/nar/gkw247.
